# Seeding Alzheimer’s disease-associated tau pathology in *MAPT* knock-in primary neurons causes early axonopathy and synaptic dysfunction

**DOI:** 10.1038/s41598-025-21920-8

**Published:** 2025-10-31

**Authors:** Rebecca L. Mueller, Benjamin Combs, Nicholas M. Kanaan

**Affiliations:** 1 Department of Translational Neuroscience, College of Human Medicine, Michigan State University, Grand Rapids, MI USA; 2 Neuroscience Program, Michigan State University, East Lansing, MI USA; 3 Grand Rapids Research Center, 400 Monroe Ave NW, Grand Rapids, MI 49503 USA

**Keywords:** Alzheimer's disease, Neurodegeneration, Cellular neuroscience, Diseases of the nervous system, Molecular neuroscience, Synaptic transmission, Protein aggregation, Mechanisms of disease, Protein aggregation, Post-translational modifications, Phosphorylation

## Abstract

The progressive accumulation of pathological tau is a hallmark of Alzheimer’s disease (AD). The bulk of existing *in vitro* and *in vivo* evidence suggests that pathological tau forms can seed further aggregation of the protein. However, many of the subsequent functional consequences following the formation of pathogenic tau aggregates are not yet fully understood. Here, we utilized the tau seeding phenomenon to induce the formation of pathogenic tau and identify intracellular consequences in a neuron culture model of AD-associated tauopathy. Primary neurons from human tau knock-in (MAPT-KI) mice were seeded with human AD brain-derived insoluble tau (AD-tau). Microscopy and biochemical assays were used to characterize the pathological tau species formed, as well as the extent of neuronal, axonal and synaptic degeneration in seeded MAPT-KI neurons. In addition, high-density microelectrode arrays were used to assess synaptic functionality in seeded MAPT-KI neuron cultures. Human-derived AD-tau seeded intracellular endogenous tau inclusions that contained AD-associated modifications (i.e. phosphorylation at the PHF1, AT8, and pS422 antibody epitopes) and adopted multiple pathogenic conformations (i.e. oligomers and exposure of an N-terminal phosphatase activating domain; PAD). Tau inclusions, containing pS422 + and PAD-exposed tau, colocalized with active glycogen synthase kinase 3β (the kinase involved in PAD-mediated axonal transport impairment) and accumulations of axonal transport cargo proteins (i.e. synaptophysin and amyloid precursor protein) in dystrophic axons. While there was no overt axonal degeneration or cell loss, intact excitatory synapses were reduced in the AD-tau neurons. Neuron cultures treated with AD-tau exhibited an N-methyl-D-aspartate receptor-dependent increase in network burst frequency when activated with glutamate as measured through high-density microelectrode arrays. Together, the data demonstrate that the AD-tau seeded MAPT-KI neuron model exhibits features associated with neuronal dysfunction resembling those that occur early in human disease (i.e. axonal pathology and dystrophy, hyperexcitability and hypersynchrony), without causing overt neurodegeneration.

**Supplementary Information:**

The online version contains supplementary material available at 10.1038/s41598-025-21920-8.

## Introduction

Alzheimer’s disease (AD) is characterized by the progressive intracellular accumulation of pathologically modified tau protein. In AD, pathological tau species are comprised of tau isoforms containing three microtubule binding repeats (MTBR) (3R) and four MTBRs (4R) and are associated with specific phosphorylation patterns and conformations^1^. The pathological modifications and conformations of tau are thought to underlie tau’s ability to cause neuron dysfunction and degeneration, but precisely how these forms of tau are linked to neurodegeneration is only partially understood.

Exposing neurons to pathological tau-seeding materials, typically derived by isolating insoluble tau fractions from human diseased brains, is one common method used to induce tau accumulation in mice^2–11^ and primary neurons^4,5,10,12^. These models show that neurons take up human brain-derived pathological tau which then recruits endogenous tau into inclusions that share characteristics observed in the human AD brain (e.g. phosphorylation at specific sites and conformational modifications). Many previous seeding studies utilized transgenic mice overexpressing human tau^2–4^, including models with tau mutations^2,4,6,8^ or wildtype mice^5,7,10^. Despite advances in our understanding of the mechanisms underlying tau seeding, there is still a need to elucidate how the accumulation of intracellular tau inclusions affects neuronal integrity and function.

Evidence of synapse loss^13–16^ and axonal abnormalities^17–20^ is observed in early AD tissue, and these features are recapitulated in tauopathy mouse models^21–27^, highlighting a role for tau in the degenerative process. Tau is observed in axonal swellings alongside accumulations of synaptophysin and amyloid precursor protein (APP) in human AD tissue^18,20^, which is thought to indicate axonal dysfunction (e.g. transport deficits). Furthermore, several forms of pathological tau are linked directly to axonal transport deficits through exposure of an N-terminal phosphatase activating domain (PAD)^28–34^. Imaging studies in living patients provide evidence of neuronal hyperactivity and hypersynchrony in early stages of AD^35,36^, and animal studies have linked tau to neuronal hyperexcitability^37–41^. A model combining physiological expression of all six human tau isoforms and human AD brain-derived tau seeding material should provide an excellent model to continue studying these and other downstream consequences of pathological tau accumulation.

Here, we treated primary hippocampal neurons with AD brain-derived insoluble tau to assess how intracellular disease-relevant tau species affect neuronal viability and function. We used human tau knock-in (MAPT-KI) mice^11,42^, in which the entire murine *Mapt* gene was replaced with human *MAPT*, to overcome the inherent issues with transgenic models (e.g. tau overexpression, gene disruption, etc.) and to incorporate expression of endogenous human 3R and 4R tau isoforms. The seeded cultures exhibited the adoption of several pathogenic forms of tau, including abnormal conformations and specific phosphorylation changes, that are linked to toxicity and present in early human disease tissue. Some of these changes were previously reported and others are novel to this study. Seeded neurons also displayed early signs of axonal degeneration and accumulation of proteins in axonal swellings that suggests axonal transport deficits. Furthermore, seeded cultures showed network hypersynchrony and synapse loss. Overt cell loss was not observed, suggesting that this model recapitulates the early changes observed in AD that may precede overt neurodegeneration.

## Methods

### Animals

Breeding pairs of MAPT-KI^11^ mice were obtained from Dr. Karen Duff at Columbia University with permission from the Saido group at the Riken Center for Brain Science. In these mice, murine *Mapt* was replaced with human *MAPT* and adult MAPT-KI mice express all six human tau isoforms^11,42^. Tau knockout mice (Tau-KO; B6.129 × 1-Mapttm1Hnd/J; Jackson Labs, 007251) were used for control experiments. All mice were housed in 12 h light/dark conditions with food and water provided *ad libitum*. Timed pregnant MAPT-KI or Tau-KO females were used to obtain fetal tissue; embryonic day 0 (E0) refers to the day the vaginal plug was found. On the day of primary neuron harvest, pregnant females were euthanized by sodium pentobarbital overdose (at least 100 mg/kg) administered via intraperitoneal injection. All studies were conducted in compliance with federal, state, and institutional guidelines and with the approval of Michigan State University’s Institutional Animal Care and Use Committee. This study is reported in compliance with ARRIVE guidelines.

### Primary neuron cultures

In preparation for primary neuron harvests, glass-bottom chamber slides were coated with 0.5 mg/mL poly-D-lysine (Sigma, P7886-100MG) in borate buffer [12.5 mM sodium borate decahydrate (VWR, MK745706) and 50 mM boric acid (Sigma, B6768-1KG)] overnight at room temperature, then washed 4x with sterile deionized water. Embryonic day 16 (E16) or 18 (E18) pups were obtained from timed pregnant MAPT-KI mice or Tau-KO mice, respectively. Hippocampi from one litter (~ 5–10 pups) were dissected, pooled (each biological replicate represents cultures from a single litter) and dissociated as described previously^43^. Cells were plated in neurobasal Plus media (NBM+) supplemented with 2% B27 Plus (Gibco, A3582901), 1% GlutaMAX (Gibco, 35050061), and antibiotics amphotericin B (2.5 µg/mL; Gibco, 15290-026) and gentamicin (50 µg/mL; Gibco, 15710-072). Plating densities were as follows: 25,000 cells/well in 100 µl/well for 18-well chamber slides (Ibidi, 81817) and poly-D-lysine coated 96-well plates (Corning, 354461), or 150,000 cells/well in 500 µl/well for poly-D-lysine coated 24-well plates (Fisher, 08-774-124). Cells were maintained in a humidity-controlled incubator at 37 °C, and 5% CO_2_. Fresh NBM + was added to cells every other day: 5 µl (18-well chamber slides and 96-well plates) or 75 µl (24-well plates) per well.

### Western blotting culture lysates

Immunoblotting was used to determine the level of tau isoform expression over time in the MAPT-KI primary hippocampal neurons. Cells were collected in cell lysis buffer (20 mM Tris, 0.5 mM dithiothreitol, 150 mM NaCl, 0.5% Triton X-100) containing protease inhibitors (2 µg/mL pepstatin, 2 µg/mL bestatin, 2 µg/mL leupeptin). Lysates were sonicated and total protein was quantified using the Bio-Rad protein assay (Bio-Rad, 5000006) as directed. Next, 27.5 µL of each lysate [~ 18–46 µg; collected on day in vitro (DIV) 5, 14, 22 or 31] were combined with fresh protease inhibitors, as above, and the samples were dephosphorylated in the presence of FastAP Thermosensitive Alkaline Phosphatase (1:5; Fermentas, EF0652) at 37 °C for 3 h. Then, lysates were heated with Laemmli sample buffer (final 1X composition: 20 mM Tris, pH 6.8, 2% sodium dodecyl sulfate (SDS), 6% glycerol, 1% β-mercaptoethanol, 0.002% bromophenol blue) at 95 °C for 5 min. Samples were loaded onto 12-well 10% Tris-Glycine Plus Midi Gels (Invitrogen, WXP01012BOX) alongside 1.75 µl of All Blue Precision Plus Protein Standards (Bio-Rad, 161–0373) and 3 µl of a human tau ladder containing all six isoforms (25 ng of each isoform; rPeptide, T-1007-2) and separated by SDS-polyacrylamide gel electrophoresis at 250 V for 40 min. Proteins were transferred onto nitrocellulose membranes (Pall Life Sciences, Port Washington, NY, #66593) using a Bio-Rad transfer unit at 400 mA for 50 min. Membranes were blocked in 2% non-fat dry milk (NFDM) diluted in tris buffered saline (TBS; 500 mM Tris, 150 mM NaCl pH 7.4) for 1 h, then probed with a total tau antibody (Tau12 antibody; 1:200,000; mouse IgG1; Kanaan Lab;^44,45)^ and a neuron-specific β-III tubulin antibody (Tuj1 antibody; 1:10,000; mouse IgG2a;^46^) overnight at 4 °C. Next, membranes were washed 3 × 5 min with TBS with 0.1% TWEEN 20, then probed for 1 h with secondary antibodies (each 1:20,000) IRDye 680LT goat anti-mouse IgG1 (LI-COR Biosciences, 926-68050, RRID: AB_2783642) and IRDye 800CW goat anti-mouse IgG2a (LI-COR Biosciences, 926-32351, RRID: AB_2782998) in 2% NFDM. After secondary antibody labeling, the membranes were washed and then imaged using a LI-COR Odyssey infrared imaging system. LI-COR ImageStudioLite 5.2 Software was used to quantify the signal intensity of the bands. Signal intensities for Tau12-positive bands were reported unnormalized or normalized to Tuj1-positive bands as indicated.

### Immunocytofluorescence (ICF) to characterize cell types in MAPT-KI cultures

The cell types present in the MAPT-KI cultures were characterized using ICF with neuron, astrocyte and oligodendrocyte markers. Cells were fixed on DIV21 with warm 4% paraformaldehyde in fixation buffer [10 mM 2-(N-morpholino) ethanesulfonic acid, 138 mM KCl, 3 mM MgCl_2_, and 4 mM ethylene glycol-bis(2-aminoethylether)-N, N,N, N-tetraacetic acid, pH 6.1] for 20 min at room temperature then washed 3 × 5 min with TBS. Cells were incubated with blocking buffer (5% goat serum (GS), 1% bovine serum albumin 0.2% Triton-X in TBS) for 1 h. Then, cells were incubated with a neuronal marker (microtubule associated protein 2, MAP2 AP14 antibody; 1:10,000; mouse IgG1; Kanaan Lab; AB_2832940;^47–50^), an astrocytic marker antibody (glial acidic fibrillary protein, GFAP antibody; 1:4,000; rabbit polyclonal; Dako, Z0334; AB_10013382), and an oligodendrocyte marker antibody (myelin basic protein, MBP antibody; 1:2,000; chicken polyclonal; Thermo Fisher Scientific, PA1-10008; AB_1077024) in 2% GS-TBS overnight at 4 °C. Cells were washed 3 × 5 min with TBS, then incubated with Alexa Fluor goat anti-mouse IgG1 488 (Thermo Fisher Scientific, A21121; AB_2535764) + Alexa Fluor goat anti-rabbit 568 (Thermo Fisher Scientific, A11011; AB_143157) + Alexa Fluor goat anti-chicken 647 (Thermo Fisher Scientific, A32933; AB_2762845) secondary antibodies (all 1:500 in 2% GS-TBS) for 1 h. Cells were washed 3 × 5 min with TBS. Nuclei were labeled with 4′,6-diamidino-2-phenylindole (DAPI; 1:10,000; Thermo, D1306) in the first wash step.

### Confocal imaging

Images were obtained using a Nikon A1 + laser scanning confocal microscope system equipped with solid-state lasers (405, 488, 561, and 640 nm) and Nikon Elements AR software. All images in an experiment were acquired at 20x, 40x or 60x magnification using identical acquisition settings (i.e. gain, offset, laser intensity, pinhole size, resolution, scan speed, and step-size for z-stacks) across all experimental conditions. Maximum projections of z-stack images were used for colocalization analysis (see below) and to create figure images. Images were prepared for publication using FIJI ImageJ (version 2.14.0/1.54f), Adobe Photoshop (version 25.9.1), and Adobe Illustrator (version 28.5).

### Purification of insoluble tau from AD frontal cortex tissue

Sarkosyl-insoluble tau was purified from human AD brain frontal cortical tissue obtained from the University of Michigan Brain Bank according to methods adapted from Narasimhan and Lee^51^. Tissue from six individuals with AD (Braak stage V-VI) or age-matched cognitively unimpaired individuals (Braak stage I-II) was pooled (3.5–4.5 g total). Tissue was homogenized using a glass Dounce homogenizer in 9 volumes of ice-cold high-salt extraction buffer (10 mM Tris, pH 7.4, 10% sucrose, 0.8 M NaCl, 1 mM ethylenediaminetetraacetic acid, 0.1% sarkosyl) with protease (2 µg/mL pepstatin, 2 µg/mL bestatin, 2 µg/mL leupeptin, 4 mM phenylmethylsulfonyl fluoride, 10 µg/mL aprotinin) and phosphatase inhibitors (1 mM tetra-sodium pyrophosphate decahydrate, 10 mM β-glycerophosphate, 1 mM sodium orthovanadate, 1 M sodium fluoride). The homogenate was transferred to 50 mL conical tubes and centrifuged at 10,000xg (max) for 10 min at 4 °C in a F14-14 × 50cy fixed angle rotor (Thermo Scientific, 096-145075) and Sorvall LYNX 4000 Superspeed centrifuge (Thermo Scientific, 75006581). The supernatant was filtered through a KimWipe into a 50 mL conical tube. The pellet was homogenized and centrifuged as above. The second supernatant was filtered as above. This process was repeated one more time. The three supernatants were pooled into a glass beaker, the concentration of sarkosyl was increased to 1%, and the mixture was stirred with a stir bar on a stir plate for 1.5 h at room temperature. The suspension was transferred to 36 mL ultracentrifuge tubes (Beckman Coulter, 355618) and centrifuged at 300,000x*g* (max) at 4 °C for 60 min in a T-865 fixed angle rotor (Thermo Scientific, 51411) and Sorvall WX + Ultra centrifuge (Thermo Fisher, 75000100). The resulting pellet was washed with 1X Dulbecco’s phosphate buffered saline (dPBS; Gibco, 14200-075), broken up by pipetting, transferred to a new 36 mL tube which was then filled with dPBS, and centrifuged at 250,000x *g*(max) at 4 °C for 30 min as above. The resulting pellet was broken up in dPBS (100 µl/g of original tissue) and transferred to a 1.5 mL tube, then incubated on a shaker at room temperature overnight. The next day the pellet was homogenized by passing the suspension through a 21G needle, followed by a 27G needle and then sonicating (20 × 1 s pulses at power level 1.5; Misonix, XL-2000). The homogenate was transferred to a thickwall 0.5 mL tube (Thermo Scientific, 45235) and centrifuged at 100,000x*g* (max) at 4 °C for 30 min in a S120-AT3 rotor (Thermo Scientific, 45584) and Sorvall MTX Micro-Ultra centrifuge (Thermo Scientific, 46960). The resulting pellet was resuspended in 150 –200 µl dPBS, sonicated (60 × 1 s pulses at power level 1.5), transferred to a thickwall 0.2 mL tube (Thermo Scientific, 45233) and centrifuged at 10,000x*g* (max) for 30 min at 4 °C in a S100-AT3 fixed angle rotor (Thermo Scientific, 45585) and Sorvall MTX Micro-Ultra centrifuge. The resulting supernatant containing the sarkosyl-insoluble tau from AD (referred to as AD-tau from here on) or cognitively unimpaired (referred to as Con from here on) samples was sonicated (60 × 1 s pulses at power level 1.5), then aliquoted and stored at -80 °C until use.

### Characterization of seeding material

The human-derived AD-tau and Con samples were extensively characterized to quantify the amount of tau, presence of pathological tau species and seeding capacity (see Supplementary Methods and Supplementary Fig. 2 for details).

### Treatment of primary hippocampal cultures

Primary hippocampal cultures were treated on DIV5 with 28 nM of AD-tau (concentration determined by total tau sELISA), Con (matched to the total protein concentration of the AD-tau samples), or dPBS vehicle for all seeding experiments with the exception of the PLA experiments in which cultures were treated with 56 nM AD-tau. Groups will be referred to as PBS, Con or AD-tau to indicate treatment groups from here on. Treatments were prepared in fresh NBM + and prewarmed to 37 °C. The treatments were mixed briefly by vortex immediately before addition to cells using a spiral pattern followed by gently rocking the cultures by hand to mix. The treated cultures were returned to the incubator and maintained until cells were fixed or whole lysates collected at varying time points (DIV14–31) specified in the figure legends. The experimenter was not blinded to treatment conditions during data collection and analysis due to the obvious differences in phenotype between differently treated primary cultures. Bias was minimized by randomizing treatment well location on plates, handling samples identically and running samples from each experimental group together in assays.

### MAPT-KI seeding time course experiment

Primary neuron cultures were treated with PBS, Con, or AD-tau on DIV5. At 9d, 17d, or 26d post-treatment (corresponding to DIV14, 22, or 31), cells were fixed with ice-cold methanol at 20 °C to remove soluble tau. ICF was performed as described above using the PAD-exposed tau-specific (TNT1 antibody; 1:20,000; mouse IgG1; Kanaan Lab; AB_2736930;^29,44)^ and β-III tubulin (Tuj1 antibody; 1:5,000) primary antibodies. Reactivity was detected using Alexa Fluor goat anti-mouse IgG1 568 (Thermo Fisher Scientific, A21124; AB_2535766) and Alexa Fluor goat anti-mouse IgG2a 488 (Thermo Fisher Scientific, A21131; AB_2535771) secondary antibodies (each 1:500). Cells were stained with a nuclear counterstain (DAPI). Cells were imaged using a Lionheart FX Automated Microscope (BioTek Instruments) and analyzed by Gen5 Software (version 3.11.19). Twelve images per well were captured at 10x magnification with blue (excitation 377/emission 447), green (excitation 469/emission 525) and red (excitation 531/emission 685) filter cubes. The analysis for the β-III tubulin channel (green) was run with the following settings: pixel intensity threshold of 1000, background set to dark, split touching objects = no, fill holes in mask = no, background flattening was applied with a rolling ball diameter set to 10 μm (16 pixels), image smoothing strength was 0, evaluate background on 25% of lowest pixels, minimum and maximum object size were set to 1 μm and 10,000 μm, respectively, and primary edge objects were included. The analysis for the PAD-exposed tau (red) channel was run with the following settings: pixel intensity threshold of 15,000, background set to dark, split touching objects = yes, fill holes in mask = yes, no background flattening, image smoothing strength was 0, evaluate background on 5% of lowest pixels, minimum and maximum object size were set to 0.1 μm and 100 μm, respectively, and primary edge objects were included. The number of PAD-exposed tau inclusions (TNT1) was normalized to the staining area of neuronal β-III tubulin antibody staining (Tuj1). Data corresponding to out of focus images were excluded from the analysis.

### Trypsin extraction to confirm intracellular seeding

To remove extracellular proteins, AD-tau treated cultures were treated with 0.06% trypsin- ethylenediaminetetraacetic acid for 3 min at room temperature at DIV26 (21 days post-treatment), then immediately fixed with 4% paraformaldehyde in fixation buffer and washed as above. Cells were then used in ICF studies to determine whether the tau pathologies were intra- or extracellular.

### Multi-label ICF of PBS, Con, or AD-tau treated primary MAPT-KI neurons

The colocalization between multiple tau markers, synaptic cargoes, and active glycogen synthase kinase 3β (aGSK3β) markers were determined using ICF as described above. The following combinations of antibodies were used:

1. PAD-exposed tau (TNT1 antibody; 1:20,000) + β-III tubulin (Tuj1 antibody; 1:5,000); Alexa Fluor goat anti-mouse IgG1 568 + Alexa Fluor goat anti-mouse IgG2a 488.
2. PAD-exposed tau (TNT1 antibody; 1:20,000) + microtubule-associated protein 2 (MAP2 antibody; 1:250; Rabbit; Cell Signaling Technologies, 8707 S; AB_27222660) + β-III tubulin (Tuj1 antibody; 1:5,000); Alexa Fluor goat anti-mouse IgG1 568 + Alexa Fluor goat anti-rabbit 405 (Thermo Fisher Scientific, A48254; AB_2890548) + Alexa Fluor goat anti-mouse IgG2a 488.
3. PAD-exposed tau (TNT1 antibody; 1:20,000) + oligomeric tau (TOC1 antibody; 1:1000; mouse IgM; Kanaan Lab; AB_2832939;^52,53)^ + β-III tubulin (Tuj1 antibody; 1:5,000); Alexa Fluor goat anti-mouse IgG1 488 + Alexa Fluor goat anti-mouse IgM 568 (Thermo Fisher Scientific, A21043; AB_2535712) + Alexa Fluor goat anti-mouse IgG2a 647 (Thermo Fisher Scientific, A21241; AB_2535810).
4. PAD-exposed tau (TNT1 antibody; 1:40,000), followed by Alexa Fluor 488 goat anti-mouse IgG (H + L) AffiniPure Fab fragment secondary (1:500; Jackson ImmunoResearch, 115-547-003; AB_2338869) for 1 h, then phospho-tau (biotinylated AT8 antibody; 1:100; mouse IgG1-biotin; Thermo Scientific, MN1020B; AB_223647) + β-III tubulin (Tuj1 antibody; 1:5,000), followed by Alexa Fluor streptavidin 568 (1:1000) and DyLight goat anti-mouse IgG2a 405 (1:500).
5. PAD-exposed tau (TNT1 antibody; 1:40,000), followed by Alexa Fluor 488 goat anti-mouse IgG (H + L) AffiniPure Fab fragments for 1 h, then phospho-S422 tau (pS422 antibody; 1:1000; rabbit IgG; Abcam, ab79415; AB_1603345) + phospho-S396/S404 (biotinylated PHF1 antibody; 1:100; mouse IgG1-biotin; AB_2315150;^54)^ + β-III tubulin (Tuj1 antibody; 1:5,000), followed by Alexa Fluor goat anti-rabbit 647 (1:500; Thermo Fisher, A21244; AB_2535812), Alexa Fluor streptavidin 568 (1:1000; Thermo Fisher, S11226), and DyLight goat anti-mouse IgG2a 405 (1:500; Jackson ImmuoResearch, 115-475-206; AB_2338800). The PHF1 antibody was biotinylated using N-hydroxysuccinimide-polyethylene glycol-biotin (NHS-PEG4-Biotin; Thermo, A39259) per the manufacturer’s instructions.
6. PAD-exposed tau (TNT1 antibody; 1:40,000), followed by Alexa Fluor 594 goat anti-mouse IgG (H + L) AffiniPure Fab fragments (1:500; Jackson ImmunoResearch, 115-587-003; AB_2338900) for 1 h, then caspase 3 cleaved tau (biotinylated TauC3 antibody; 1:100; mouse IgG1; Kanaan Lab; AB_2832930;^55)^ + β-III tubulin (Tuj1 antibody; 1:5,000), followed by Alexa Fluor streptavidin 488 (1:1000) and DyLight goat anti-mouse IgG2a 405 (1:500). TauC3 was biotinylated using NHS-PEG4-Biotin per the manufacturer’s instructions.
7. Following staining with thiazine red dye (ThR; see below); PAD-exposed tau (TNT1 antibody; 1:40,000) + β-III tubulin (Tuj1 antibody; 1:5,000); Alexa Fluor goat anti-mouse IgG1 488 + Alexa Fluor goat anti-mouse IgG2a 647.
8. Phospho-S422 tau (pS422 antibody; 1:1000) + active GSK3β (aGSK3β; non-phospho-Ser9 GSK3β-specific antibody; 1:100; mouse IgG1; AB_2832942;^56)^ + β-III tubulin (Tuj1 antibody; 1:5,000); Alexa Fluor goat anti-rabbit 488 (Thermo Fisher Scientific, A32731; AB_2633280) + Alexa Fluor goat anti-mouse IgG1 568 + Alexa Fluor goat anti-mouse IgG2a 647.
9. PAD-exposed tau (TNT1 antibody; 1:40,000) + presynaptic vesicles (synaptophysin antibody; 1:100; rabbit IgG; Abcam, ab52636; AB_882786) + β-III tubulin (Tuj1 antibody; 1:5,000); Alexa Fluor goat anti-mouse IgG1 488 + Alexa Fluor goat anti-rabbit 568 (Thermo Fisher Scientific, A11036; AB_143157) + Alexa Fluor goat anti-mouse IgG2a 647.

Images were acquired with confocal microscopy as described above.

### Colocalization analysis

Colocalization was measured using confocal z-stack images described above from the AD-tau treated cultures to determine the extent to which the various markers colocalized with PAD-exposed tau (TNT1 antibody). Just Another Colocalization Plugin (JACoP)^42^ in FIJI ImageJ (version 2.14.0/1.54f) was used to measure the fraction of PAD-exposed tau (TNT1 antibody) signal that overlapped with oligomeric tau (TOC1 antibody), phospho-tau (AT8 antibody), phospho-tau (PHF1 antibody), phospho-tau (pS422 antibody), cleaved tau (TauC3 antibody), or synaptophysin (Syn antibody). The fraction of phospho-tau (pS422 antibody) that overlapped with active GSK3β (aGSK3β antibody) was also measured. The z-stacks were opened, channels were split, the JACoP was used to set thresholds for each channel (see Supplementary Table S1), and then the stacks were analyzed using the M1 colocalization coefficient (fraction of TNT1 or pS422 overlapping with the other marker). Each experiment used three z-stacks per independent experimental replicate, which were averaged to create one value for each replicate (*N* = 3 for all experiments). Values were reported as mean ±standard deviation (SD).

### Thiazine red staining and ICF

The extent to which seeded neurons contained mature filamentous tau aggregates containing β-sheet structures was assessed using thiazine red (ThR) staining. Cells were fixed in warm 4% paraformaldehyde in fixation buffer as above. Prior to ICF, cells were stained with 0.005% ThR (Sigma, S570435) in 50% ethanol/TBS for 15 min at room temperature. Cells were washed 3 × 1 min with 50% ethanol/TBS, then 3 × 5 min with TBS. Cells were then immunolabelled with PAD-exposed tau (TNT1) and β-III tubulin (Tuj1) antibodies as above. Images were acquired with confocal microscopy as described above.

### Sandwich enzyme-linked immunosorbent assays (sELISAs)

To measure total tau and the presence of pathogenic tau conformations in the PBS, Con, and AD-tau treated culture lysates, non-denaturing sELISAs were used as previously described^57^. Cell lysates were collected in cell lysis buffer (20 mM Tris, 0.5 mM dithiothreitol, 150 mM NaCl, 0.5% Triton X-100, 2 µg/mL pepstatin, 2 µg/mL bestatin, 2 µg/mL leupeptin, 4 mM phenylmethylsulfonyl fluoride, 10 µg/mL aprotinin, 1 mM tetra-sodium pyrophosphate decahydrate, 10 mM β-glycerophosphate, 1 mM sodium orthovanadate, 1 M sodium fluoride, pH 7.5). Lysates were sonicated and protein concentration quantified using the Bio-Rad protein assay as directed. The capture antibodies used were for total tau (Tau5 antibody; mouse IgG1; Kanaan Lab; AB_2721194;^58,59)^, PAD-exposed tau (TNT1 antibody), or oligomeric tau (TOC1 antibody). Lysates were adjusted to 5 µg (Tau5 assays) or 20 µg (TNT1 and TOC1 assays) in TBS. The detection antibody was a rabbit polyclonal pan tau antibody (R1 antibody; 1:10,000; Kanaan Lab; AB_2832929;^60)^. Absorbance data (an inverse log scale measure) were converted to percent light absorbed (a linear scale conversion) using the following equation where x is absorbance.

% Absorbance = (1–10^− x^) _✱_100.

### Overt cell toxicity and axonal degeneration assays

Cell toxicity was measured in PBS, Con, and AD-tau cultures using CellTiter-Glo and ApoTox-Glo assays, manual cell counts (neurons, astrocytes, and oligodendrocytes), and immunoblotting for neuronal (β-III tubulin; Tuj1 antibody) or astrocytic (GFAP antibody) protein levels. Axonal degeneration was measured indirectly using a Calpain-Glo Assay and directly using ICF and confocal microscopy image analysis, while axonal tracking with high-density microelectrode arrays (MEAs) was used for assessing axonal function. See Supplementary Methods for the details of these experiments.

### Proximity ligation assay (PLA)

Synaptic density was measured using a recently described synaptic PLA method^61^ to determine if seeding caused excitatory synaptic loss. Primary MAPT-KI neurons were plated in poly-D-lysine-coated 18-well glass-bottom chamber slides at a density of 25,000 cells/well in 100 µl of NBM+. Cultures were treated on DIV5 with PBS, Con, or AD-tau. Cells were fixed on DIV33 (28d post-treatment) with 4% paraformaldehyde, washed and blocked (5% GS, 1% bovine serum albumin, and 0.2% triton-X in TBS) as above. Then cultures were incubated overnight at 4 °C with homer 1 antibody (2 ng/µL; rabbit; Synaptic Systems, 160003; AB_887730) and bassoon antibody (2 ng/µL; mouse IgG2b; NeuroMab, 73–491; AB_2716712) diluted in 2% GS-TBS. Untreated cultures were incubated with either homer 1 (bassoon primary antibody omitted) or bassoon (homer 1 primary antibody omitted), or no antibody (both primary antibodies omitted) as controls for the PLA method using the Duolink PLA kit (Sigma-Aldrich, DUO96020). After primary antibody incubation, the cultures were washed 4 × 5 min in Buffer A, then incubated with anti-mouse PLUS and anti-rabbit MINUS secondary antibody probes (each diluted 1:15 in 2% GS-TBS) for 1 h at 37 °C. Cultures were washed 4 × 5 min in Buffer A, then incubated in ligase (diluted 1:40 in 1X ligation buffer) for 30 min at 37 °C. Cells were washed again 4 × 5 min in Buffer A, then incubated in polymerase (diluted 1:80 in 1X amplification buffer) for 100 min at 37 °C. Then, cells were washed 2 × 10 min in Wash Buffer B at room temperature. Next, cultures were incubated in blocking buffer and immunolabelled (as above) with PAD-exposed tau (TNT1 antibody; 25 ng/mL) and β-III tubulin (Tuj1 antibody; 100 ng/mL) primary antibodies, and Alexa Fluor goat anti-mouse IgG2a 568 (Thermo Fisher Scientific, A21134; AB_2535773) and goat anti-mouse IgG1 647 (Thermo Fisher Scientific, A21240; AB_2535809) secondary antibodies. DAPI nuclear counterstain (1:10,000) was included in the first wash to label nuclei.

Cells were imaged as 40x z-stacks (0.5 μm step sizes and 11 images/stack) with a Nikon A1 + laser scanning confocal microscope (as above). Maximum projection images of the z-stacks were generated in NIS-Elements. The channels were split in FIJI ImageJ (version 2.14.0/1.54f) for data quantitation. A threshold mask was generated for the PLA puncta (green channel) using the default threshold setting and the number of puncta per image were counted using the Analyze Particles function (Size: 0-infinity, Circularity: 0.00–1.00). A separate threshold mask was generated for the β-III tubulin immunolabelling (red channel) using the Huang setting and the total area of the mask was calculated using the Measure function. Synaptic density was calculated as the number of synaptic PLA puncta/total β-III tubulin positive area (µm^2^) for each image. Individual data points represent the mean of the results from all analyzed images within an individual experimental replicate, and the experiment was repeated four independent times (*N* = 4). Representative images were prepared for publication using FIJI ImageJ (version 2.14.0/1.54f), Adobe Photoshop (version 25.9.1), and Adobe Illustrator (version 28.5).

### Plating primary neurons on high-density MEAs

Two days prior to plating, 18 MaxOne Chips (MaxWell Biosystems AG MaxOne Chip MX1-S/U-CHP) were treated with 1 mL/well of 1% Terg-a-zyme (Sigma-Aldrich, Z273287) for 2 h at room temperature. Chips were washed 6x with ultra-pure diH_2_O, then sterilized via immersion in 70% ethanol for 30 min at room temperature. Chips were washed 4x with sterile ultra-pure diH_2_O and air dried. Then, each chip was filled with 600 µL of NBM Electro (NBM-E; Gibco, A14098-01) supplemented with 2% B27 Electro (Gibco, A14097-01), 1% GlutaMAX (Gibco, 35050061), and antibiotics amphotericin B (2.5 µg/mL) and gentamicin (50 µg/mL), placed into a covered petri dish containing a 50 mL conical tube cap filled with sterile water, and stored in a humidity-controlled 37 °C incubator with 5% CO_2_ for two days. On the day of the primary neuron harvest, each well was washed 1x with sterile water and the electrode surface was coated with 50 µl of 0.1 mg/mL poly-D-lysine (Sigma, P7886) in borate buffer for 2 h at 37 °C. Then, each well was washed 4x with sterile water and air dried for 45 min. Next, the electrode surface was coated with 50 µl of 0.02 mg/mL laminin (Thermo Fisher, 23017015) for 1 h at 37 °C. During the coating steps, primary hippocampal neurons were obtained from E16 MAPT-KI mice (pooled from one timed pregnant female) as described above. Immediately prior to the addition of cells, the laminin was removed and then a 50 µl droplet of 100,000 cells in NBM-E was plated onto the electrode surface. The cells settled onto the chip for 1 h at 37 °C and then 350 µl of NBM-E/well was added. Cultures received 80 µl of fresh NBM-E/well 3x per week. Cultures were treated at DIV5 with PBS, Con or 28 nM AD-tau (6 chips/treatment).

### MEA culture activity recordings and data analysis

On DIV28-29, active electrodes (i.e. electrodes at which action potentials were detected) in the MEAs were identified using an ActivityScan Assay with the following parameters: checkerboard configuration; 12,980 electrodes recorded; 30 s per configuration. The gain was set to 512x with a ~ 300 Hz high pass filter and a spike threshold of 5.00 for all recordings (MaxLab Live software version 24.2.4). Each culture received 80 µl of fresh NBM-E 1 h prior to recording. The chip was connected to the recording unit in the incubator and allowed to equilibrate for 10 min prior to obtaining the neuronal activity scan recordings.

Immediately following the ActivityScan Assay, a Network Assay was performed to obtain the culture network activity using ~ 1020 randomly distributed active electrodes (as determined from the ActivityScan). Each chip had a unique electrode configuration that was saved and used for the subsequent network recordings. Culture network activity within each culture was recorded at baseline for 5 min. Immediately following the baseline network activity recording, the culture was treated with L-glutamic acid (Glu; 20 µM final concentration; Sigma, G1251-1G), a glutamate receptor agonist, and then the chip was gently rocked 5x to mix and a 5 min network activity recording was performed using the same NetworkAssay parameters as the baseline recording. Immediately following the Glu recordings, the culture was treated with (2R)-amino-5-phosphonopentanoate (AP5; 5 µM final concentration; Tocris, 0106), an N-methyl-D-aspartate receptor (NMDAR) antagonist, as above and then a 5 min network activity recording was obtained using the same Network Assay parameters as above.

For the network burst frequency analysis, a Network Analysis was run with a smoothing window size of 0.3 s, a minimum peak distance of 1.0 s, a start-stop threshold of 0.3, and a burst detection threshold of 1.5 Hz. Data from MEA experiments were handled three ways. First, network burst frequency values (Hz) are reported and used for statistical comparison. Second, burst frequency data were normalized to the mean baseline values within each group to illustrate change from baseline in response Glu or AP5 treatment. Third, network burst frequency data following Glu treatment were normalized to baselines for each independent replicate, and data following AP5 treatment were normalized to Glu values for each independent replicate and used for statistical comparisons. The MEAs were also used to measure mean firing rates (see Supplementary Methods for details). After completing the final recording culture lysates were collected to confirm the presence of PAD-exposed and oligomeric tau using sELISAs (see Supplementary Methods for details).

### Statistical analyses

Each independent experimental replicate (*N* = 1) represents a single primary cell culture obtained by pooling hippocampal tissue from ~ 5–10 fetuses from one timed pregnant MAPT-KI or Tau-KO female mouse. For the synaptic MEA experiments, each experimental replicate (*N* = 1) represents a single MaxOne MEA chip. Primary MAPT-KI neurons from a single harvest (one timed pregnant MAPT-KI female) were plated onto 18 MaxOne chips (6 per treatment group). Experiments that underwent data and statistical analysis each had three treatment groups (PBS, Con, and AD-tau), and each experiment had a sample size of three or more independent replicates (as indicated in the figure legends). The data were assessed for normality and equal variance using the Shapiro-Wilk normality test and the Brown-Forsythe variance test. Experiments that met normality and equal variance tests were analyzed with a one-way analysis of variance (ANOVA) test followed by Tukey’s post hoc test when overall significance was achieved. When both were not met, the Kruskal-Wallis nonparametric test was used followed by the Dunn’s post hoc test when overall significance was achieved. Significance was defined as *p* ≤ 0.05. Data are shown as mean ± SD for parametric tests or as median ± interquartile range for nonparametric tests (as indicated in figure legends). Analyses were performed using GraphPad Prism 10 software (version 10.2.3).

## Results

### Primary MAPT-KI culture characterization

**Fig. 1:**
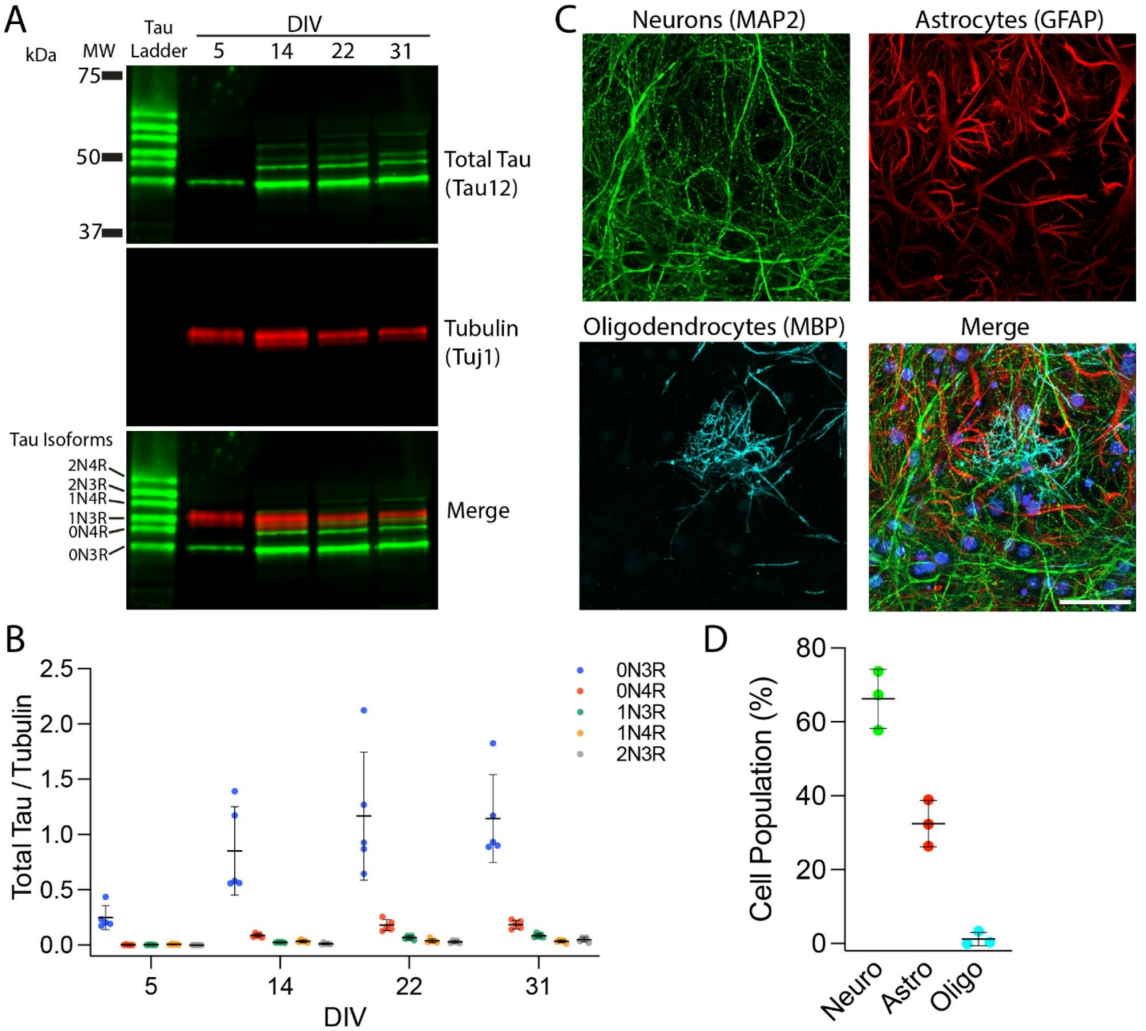
Primary hippocampal cultures from MAPT-KI mice express multiple 4R and 3R human tau isoforms and are comprised of neurons, astrocytes and oligodendrocytes. (**A**) Representative Western blot of MAPT-KI primary culture lysates collected on DIV5, 14, 22, and 31, probed for total tau (Tau12 antibody; green) and β-III tubulin (Tuj1 antibody; red). Note that the position of each isoform (2N4R, 2N3R, 1N4R, 1N3R, 0N4R and 0N3R) is shown with a recombinant human tau ladder. Uncropped versions of the representative blot are shown in Supplementary Fig. 14A. (**B**) The intensity of each isoform band was quantified and normalized to β-III tubulin signal (data are mean ±SD). *N* = 5. Note, graphs of normalized signal for each individual isoform are shown in Supplementary Fig. 1B-F, and the unnormalized signal for all isoforms or individual isoforms are reported in Supplementary Fig. 1G or H-L, respectively. (**C**) Primary cultures (DIV21) were immunolabelled with MAP2 (neurons; green), GFAP (astrocytes; red), and MBP (oligodendrocytes; cyan) antibodies; DAPI (nuclei: blue) is shown in the merged image. Scale bar = 50 μm. (**D**) Neurons (Neuro), astrocytes (Astro) and oligodendrocytes (Oligo) were manually counted and divided by the total cell number (% cell population; data are mean ±SD). *N* = 3.

The MAPT-KI mice were previously generated by replacing the entire mouse *Mapt* gene with human *MAPT* and express all six human tau isoforms as adults^11,42^. In contrast to adult expression patterns, only 3R tau is expressed during development in humans and mice^62–64^. The relative expression levels of the six human tau isoforms in MAPT-KI primary neuron cultures were not investigated previously. To address this, we quantified the protein expression levels of each tau isoform in MAPT-KI primary cultures collected on DIV5, 14, 22 and 31 using Western blots. The 0N3R tau isoform was the predominantly expressed isoform from DIV5-31 and the only isoform detected at DIV5 (Fig. 1A, B and Supplementary Fig. 1). At DIV14, 22 and 31 we detected the 0N4R, 1N3R, 1N4R, and 2N3R isoforms (Fig. 1A, B and Supplementary Fig. 1). The 2N4R isoform was the only major isoform that was not detectable in the lysates at the time points analyzed (Fig. 1A, B and Supplementary Fig. 1). Quantification of the tau signal alone (i.e.; without β-III tubulin normalization) is provided in Supplementary Fig. 1G-L).

Using microscopy, we characterized the cell-types present in DIV21 MAPT-KI cultures by triple-label ICF with a neuron-specific MAP2 antibody, an astrocyte-specific GFAP antibody, and an oligodendrocyte-specific MBP antibody (Fig. 1C). Each cell type was manually counted. The cultures were composed primarily of neurons (~ 66%), with a substantial astrocyte component (~ 33%) and a small oligodendrocyte component (~ 1%) (Fig. 1D).

### Seeding material contains pathological tau species

Using a sarkosyl-based purification protocol, we generated samples enriched for sarkosyl-insoluble tau (AD-tau) from post-mortem late-stage AD (Braak stage V-VI) human frontal cortex tissue. To control for other sarkosyl-insoluble proteins, we also produced samples from cognitively intact age-matched individuals (Con, Braak stage I-II) using the same purification protocol. In our hands, Con and AD-tau preparations contained 0.07–0.64% or 3.6–11.4% tau, respectively (see Supplementary Methods). We validated our samples by confirming that AD-tau, but not the Con samples, contained detectable paired helical filaments (Supplementary Fig. 2A), tau phosphorylated at S396/S404 (PHF1-reactive; Supplementary Fig. 2B), 3R and 4R tau isoforms (Supplementary Fig. 2C), the pathogenic PAD-exposed conformation (TNT1-reactive, Supplementary Fig. 2E), and tau oligomers (TOC1-reactive; Supplementary Fig. 2F).

We verified the seeding capacity of the brain-derived samples using the HEK293 RD Biosensor cells that express fluorescently tagged P301S mutant MTBR proteins^65^. After AD-tau treatment, bright inclusions composed of fluorescent protein-tagged MTBR protein were observed (Supplementary Fig. 2G). Only a small amount of seeding was seen in the cells treated with Con sample (Supplementary Fig. 2G), which is consistent with substantially lower levels of tau pathology in Braak I-II brains. Together, these results show that AD-tau samples contain known AD-associated pathological forms of tau capable of seeding tau inclusions in cultured biosensor cells.

### Treatment of MAPT-KI neurons with AD-tau results in the progressive formation of PAD-exposed tau inclusions

**Fig. 2:**
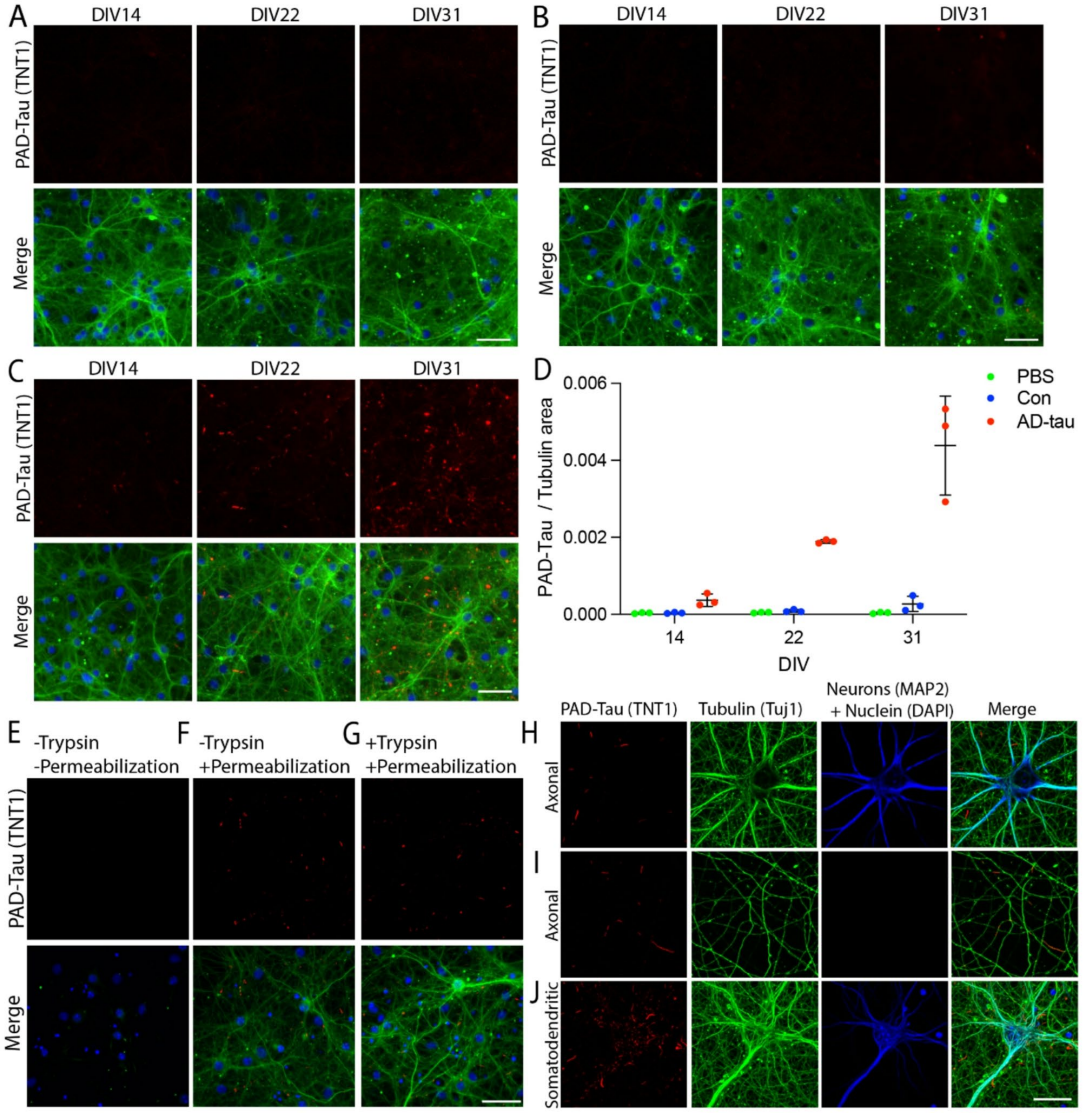
Progressive formation of intracellular PAD-exposed tau inclusions in MAPT-KI primary neurons treated with AD-tau. (**A**–**C**) PAD-exposed tau (PAD-Tau; TNT1 antibody; red) showed a progressive increase from 14–31 days in vitro (DIV) in neurons treated with AD-tau (**C**) and Con (**B**) samples at DIV5, but not PBS control cultures. The merged images include staining for PAD-Tau, β-III tubulin (Tuj1 antibody; green), and a nuclear counterstain (DAPI; blue). Scale bar = 50 μm. D) Quantitation of the number of PAD-exposed tau inclusions (PAD-Tau) normalized to the β-III tubulin staining area (data are mean ±SD). (**E**–**G**) Non-permeabilized (**E**) and permeabilized (**F**) cultures (treated at DIV5 and collected at DIV26) were immunostained for PAD-exposed tau (PAD-Tau; red). Omission of the permeabilization step showed no PAD-exposed tau, indicating that the inclusions were intracellular. (**G**) Prior to fixation and permeabilization, cultures were treated with trypsin to remove extracellular proteins and then immunostained. PAD exposed tau inclusions (PAD-Tau; red) were observed in the trypsin-treated cells, confirming that the inclusions were intracellular. The merged images show PAD-Tau, β-III tubulin (Tuj1 antibody; green) and a nuclear counterstain (DAPI; blue). Scale bar = 50 μm. (**H**–**J**) PAD-exposed tau inclusions (PAD-Tau; red) were mostly restricted to the axons of neurons (**H** and **I**), as indicated by processes that are MAP2-negative (somatodendritic marker; blue) and β-III tubulin-positive (somatodendritic and axon marker; green). Only rare localization was seen in the somatodendritic compartment of neurons (**J**), as indicated by processes being MAP2-positive and β-III tubulin-positive. DAPI was included as a nuclear counterstain (blue). Scale bar = 25 μm. *N* = 3.

We performed a seeding time-course experiment in MAPT-KI primary hippocampal neurons and determined that AD-tau seeds the formation of PAD-exposed tau conformations, which are relevant to tauopathies and directly linked to mechanisms of tau-mediated toxicity^28–34,44,66,67^. Immunostaining with the conformation-specific antibody for PAD-exposed tau (TNT1) and the neuronal β-III tubulin (Tuj1) antibody (Fig. 2A–C) demonstrated a progressive increase in PAD-exposed tau inclusions over time (DIV 14-31) in AD-tau treated neurons (Fig. 2C and D). Specifically, the PAD-exposed tau inclusion load increased ~ 5-fold from DIV14 to 22, ~ 2-fold from DIV22 to 31 and ~ 12-fold from DIV14 to 31 (Fig. 2D). The Con treated cultures developed a small number of PAD-exposed tau inclusions that increased ~ 2-fold from DIV14 to 22, ~ 3-fold from DIV22 to 31 and ~ 8-fold from DIV14 to 31 (Fig. 2D). Notably, the PAD-exposed tau inclusion load was ~ 16x greater in the AD-tau cultures compared to the Con cultures at DIV31. To confirm that the tau inclusions observed in the MAPT-KI cultures resulted from seeding endogenous tau, we treated Tau-KO primary cultures with PBS, Con, or AD-tau (28nM) on DIV5 and observed no PAD-exposed tau pathology by DIV33 (Supplementary Fig. 3A–C).

We observed that preventing antibody access to intracellular epitopes by omitting the permeabilization step in our ICF protocol resulted in the loss of PAD-exposed tau antibody (TNT1) reactivity in MAPT-KI cultures treated with AD-tau (Fig. 2E), confirming that the PAD-exposed tau inclusions observed in the permeabilized cells (Fig. 2F) were intracellular. Furthermore, AD-tau treated cultures that were treated with trypsin before fixation to remove all extracellular proteins retained PAD-exposed tau inclusions (Fig. 2G). Together with the time-course study, these data provide strong evidence that the inclusions observed in the AD-tau treated cultures are formed within neurons through the seeding of endogenous tau and they adopt PAD-exposed pathogenic conformations.

### PAD-exposed tau inclusions are primarily found in axons

To determine the cell type and subcellular localization of the PAD-exposed tau inclusions, we treated MAPT-KI cultures and co-stained for PAD-exposed tau and cell type-specific markers. Using MAP2 to label the somatodendritic compartment of neurons and β-III tubulin (Tuj1 antibody) to label the somatodendritic compartment and axons in DIV21 cultures, we confirmed that the PAD-exposed tau inclusions primarily localized to neuronal axons (MAP2–/Tuj1 + processes; Fig. 2H, I) with rare somatodendritic localization (MAP2+/Tuj1 + processes; Fig. 2J). We did not identify PAD-exposed tau inclusions in GFAP-positive astrocytes (Supplementary Fig. 3D).

### PAD-exposed tau inclusions colocalize with AD-associated oligomeric tau species

**Fig. 3:**
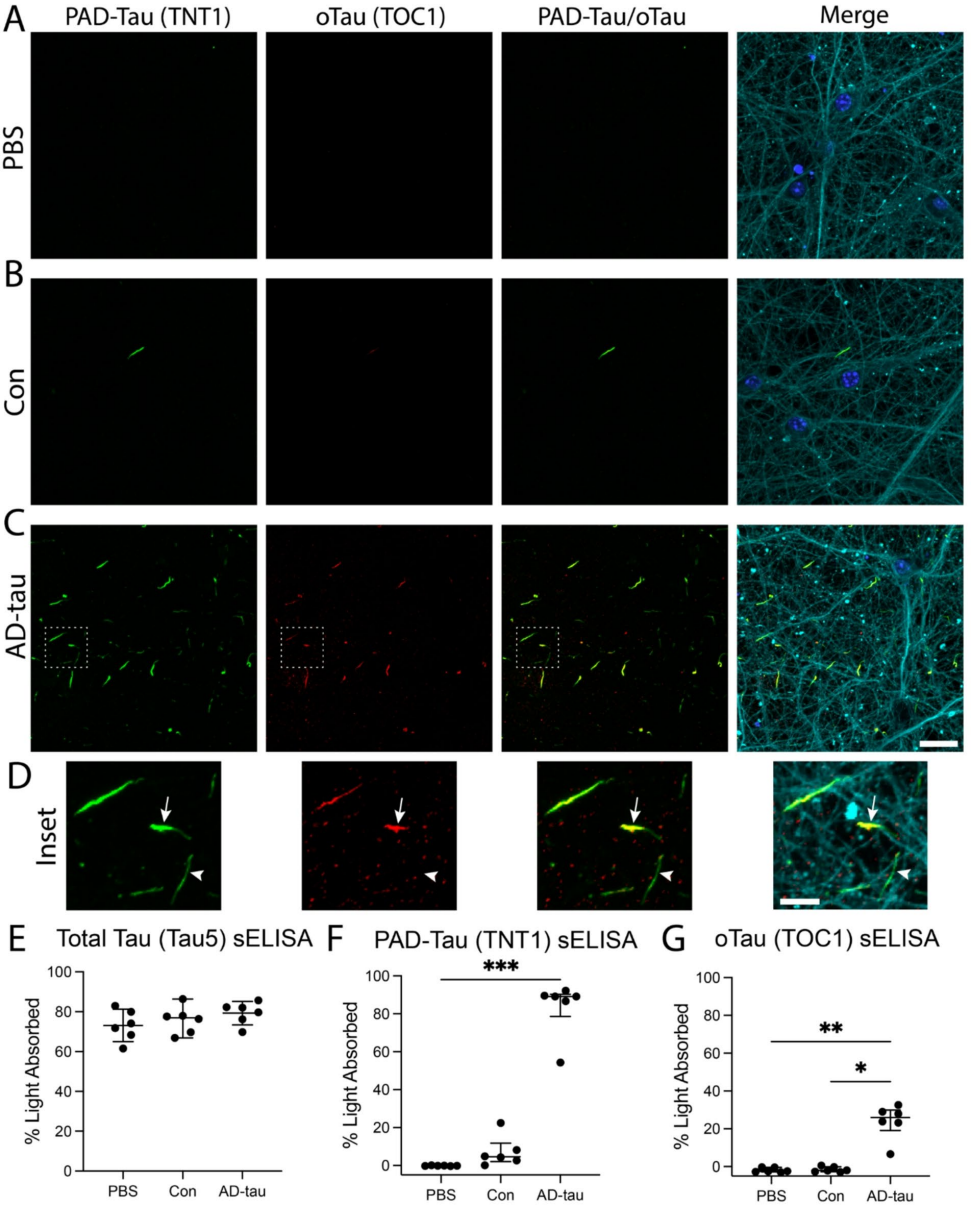
Seeded tau inclusions are comprised of PAD-exposed and oligomeric tau species. MAPT-KI cells were treated with PBS, Con, or AD-tau on DIV5 and fixed on DIV21. (**A**–**C**) PAD-exposed tau inclusions (PAD-Tau; TNT1 antibody; green) were colocalized with oligomeric tau (oTau; TOC1 antibody; red) in AD-tau (**C**) and Con (**B**) treated neurons, but not in PBS treated neurons (**A**). The merged images include PAD-Tau, β-III tubulin (Tuj1 antibody; cyan) and a nuclear counterstain (DAPI; blue). Scale bar = 20 μm. (**D**) Insets show colocalization between PAD-tau and oTau (arrow) and some inclusions with PAD-tau but not oTau (arrowhead). Scale bar = 5 μm. (**E**–**G**) Sandwich ELISAs (sELISAs) for total tau (**E**; Tau5 antibody capture), PAD-exposed tau (**F**; TNT1 antibody capture), and oligomeric tau (**G**; TOC1 antibody capture) show significant accumulation of AD-tau treated cultures at DIV31 compared to PBS treated cultures. There was a lower level of PAD-exposed tau accumulation in Con treated cultures, but this was not statistically different from PBS treated cultures. The data in E are mean ± SD and were compared using one-way ANOVA with Tukey’s multiple comparisons test. The data in F and G are median ± interquartile range and were compared using Kruskal-Wallis ANOVA with Dunn’s multiple comparisons test. *p ≤ 0.05, **p ≤ 0.01, ***p ≤ 0.001. *N* = 6.

PAD-exposed tau (TNT1 antibody) colocalized with oligomeric tau (TOC1 antibody) in cultures treated with AD-tau (Fig. 3C, D; fraction of TNT1 overlapping with TOC1 = 0.287 ±0.131) and Con-treated neurons (Fig. 3B), but both were absent in PBS-treated neurons (Fig. 3A). Primary delete stains confirmed there was no non-specific cross-reactivity between TNT1 and TOC1 antibodies (Supplementary Fig. 4). Using sELISA, we detected similar levels of total tau (Tau5 capture antibody; Fig. 3E) in the PBS, Con, and AD-tau culture lysates (one-way ANOVA; *F*_(2,15)_ = 0.2749, *p* = 0.3180), but significantly higher levels of PAD-exposed tau (TNT1 capture antibody; Fig. 3F; Kruskal-Wallis ANOVA with Dunn’s post hoc; H = 15.16, *p* < 0.0001; PBS vs. AD-tau *p* = 0.0003) and oligomeric tau (TOC1 capture antibody; Fig. 3G; Kruskal-Wallis ANOVA with Dunn’s post hoc; H = 11.66, *p* = 0.0003; PBS vs. AD-tau *p* = 0.0043; Con vs. AD-tau *p* = 0.0242) in the AD-tau culture lysates. We also detected low levels of elevated PAD-exposed tau in the Con culture lysates (Fig. 3F), but without statistically significant differences compared to PBS cultures.

### PAD-exposed tau inclusions colocalize with AD-associated phospho-tau species

**Fig. 4:**
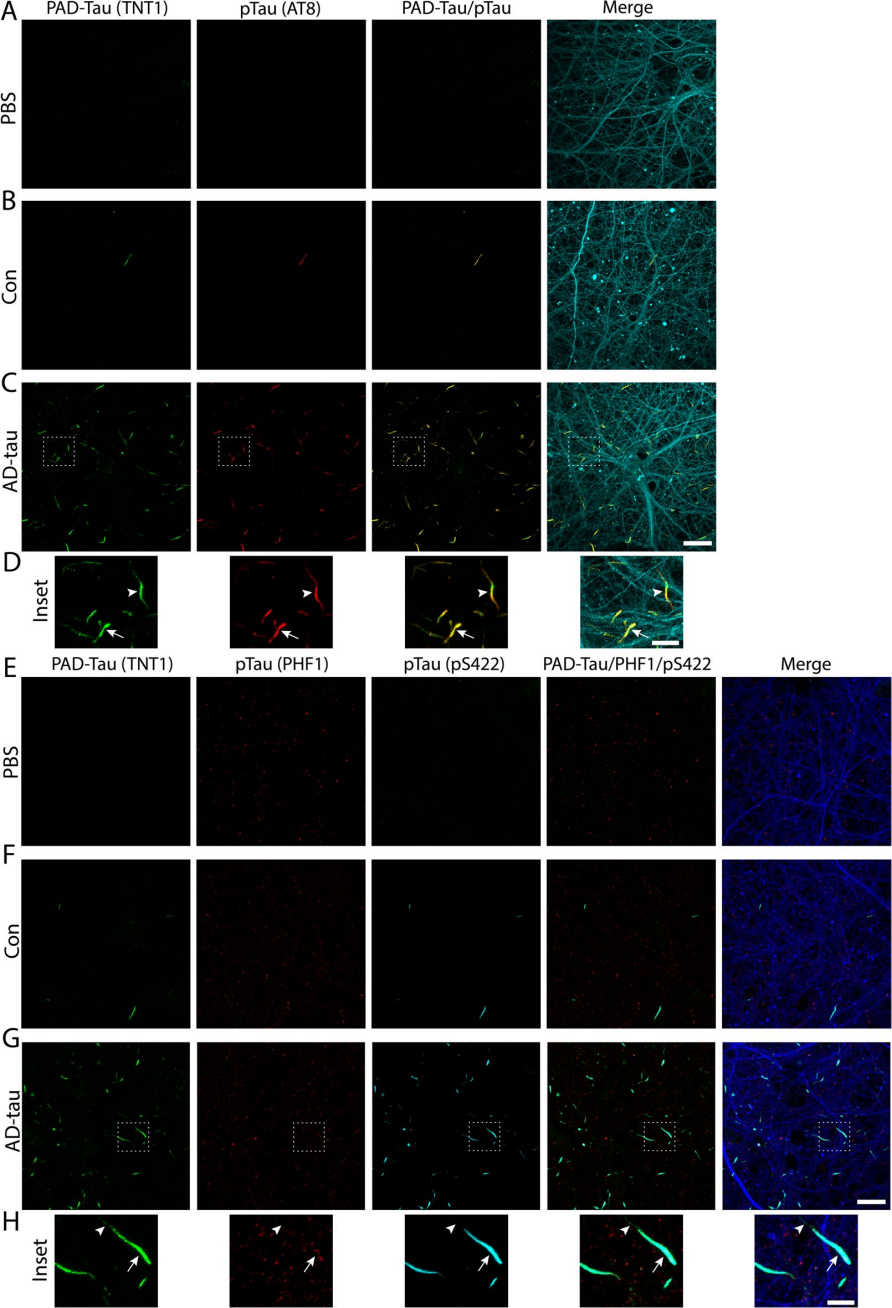
PAD-exposed tau inclusions contain phosphorylated tau. MAPT-KI cultures were treated with PBS, Con, or AD-tau on DIV5 and fixed on DIV21. (**A**–**C**) PAD-exposed tau inclusions (PAD-Tau; TNT1 antibody; green) showed extensive colocalization with AT8 phosphorylated tau (pTau; AT8 antibody; red) in AD-tau (**C**) and Con (**B**) treated neurons, not PBS treated cultures (**A**). The merged images include PAD-Tau, AT8 pTau and β-III tubulin (Tuj1 antibody; cyan). Scale bar = 20 μm. (**D**) Insets show colocalization between PAD-tau and pTau (AT8, arrow) and some inclusions with less colocalization between PAD-tau and pTau (AT8, arrowhead). Scale bar = 5 μm. (**E**–**G**) PAD-exposed tau inclusions (PAD-Tau; green) showed extensive colocalization with tau phosphorylated at Ser422 (pTau; pS422 antibody; cyan) in AD-tau (**G**) and Con (**F**) treated cultures but are not present in PBS treated cultures (**E**). Tau phosphorylated at S396/S404 (pTau; PHF1 antibody; red) showed low colocalization with the PAD-Tau and was similarly present in PBS (**E**), Con (**F**) and AD-tau (**G**) treated cultures. The merged images include PAD-Tau, PHF1 pTau, pS422 pTau, and β-III tubulin (Tuj1 antibody; blue). Scale bar = 20 μm. *N* = 3. (**H**) Insets show extensive colocalization between PAD-tau and pTau (pS422, arrow) with discrete areas of PAD-tau not colocalizing with pTau (pS422). Note, pTau (PHF1) did not show robust colocalization. Scale bar = 5 μm.

To further characterize the pathological tau species formed in the treated MAPT-KI hippocampal neurons, we assessed multiple phospho-tau antibodies for their colocalization with PAD-exposed tau. PAD-exposed tau inclusions (TNT1 antibody) were highly colocalized with AT8 phosphorylated tau (AT8 antibody; Fig. 4A–D; fraction of TNT1 overlapping with AT8 = 0.688 ±0.016) and tau phosphorylated at Ser422 (pS422 antibody; Fig. 4E-H; fraction of TNT1 overlapping with pS422 = 0.889 ±0.022) in AD-tau neurons. These markers also colocalized in the PAD-exposed inclusions in Con neurons but were absent in PBS neurons. Lower levels of colocalization were seen between PAD-exposed tau inclusions and tau phosphorylated at S396/S404 (PHF1 antibody; fraction of TNT1 overlapping with PHF1 = 0.216 ±0.076) in AD-tau neurons, and PHF1 reactivity was found in all conditions (Fig. 4E-H). Primary delete stains confirmed there was no non-specific cross-reactivity between TNT1 and AT8, pS422 or PHF1 antibodies (Supplementary Fig. 5). Together, these data indicate that treatment of MAPT-KI cultures with AD-tau results in the formation of a known early pathological tau conformation (i.e. PAD exposure) and phosphorylation of tau at multiple disease-associated sites in neurons.

### AD-tau treated neurons accumulate low levels of intermediate-stage tau species, not mature tau inclusions

**Fig. 5:**
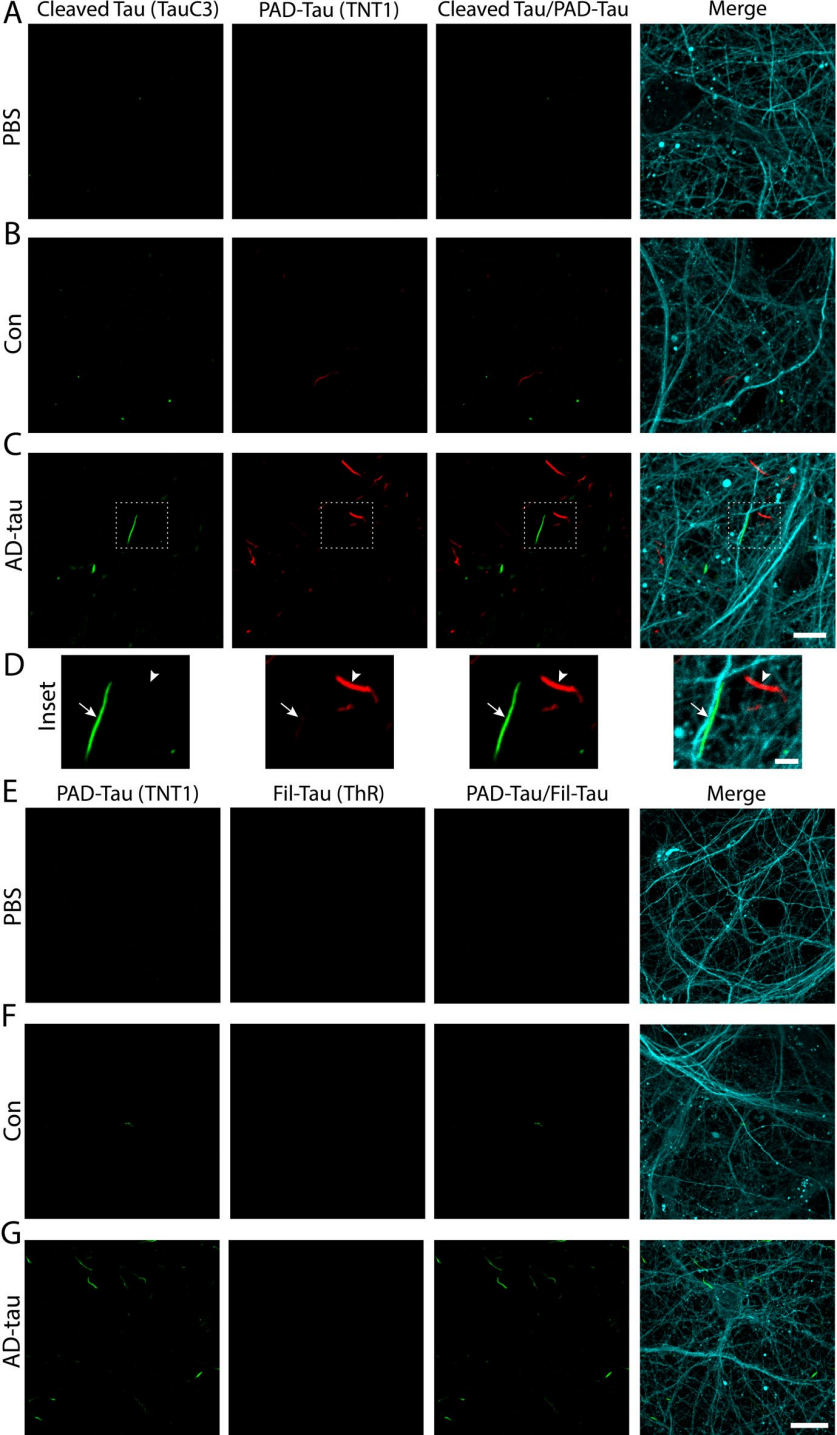
Seeded neurons develop cleaved tau inclusions, but not filamentous tau inclusions. (**A**–**C**) Tau cleaved at D421 (Cleaved Tau; TauC3 antibody; green), was present, albeit in lower abundance than PAD-exposed tau (PAD-Tau; TNT1 antibody; red), in AD-tau (**C**) and Con (**B**) treated cultures, but neither form of tau was found in PBS treated neurons (**A**). The PAD-exposed and cleaved tau inclusions rarely colocalized. The merged images include PAD-Tau, cleaved tau and β-III tubulin (Tuj1 antibody; cyan). Scale bar = 20 μm. (**D**) Insets show a clear lack of colocalization between PAD-tau (arrowhead) and cleaved tau (TauC3, arrow) inclusions. Scale bar = 5 μm. (**E–G**) Filamentous tau inclusions, as indicated by the thiazine red β-sheet dye (Fil-Tau; ThR dye; red), were not detected in any condition. The merged images include PAD-Tau, Fil-Tau and β-III tubulin (Tuj1 antibody; cyan). Scale bars = 20 μm. *N* = 3.

Having observed early pretangle changes (PAD-exposure, oligomerization and specific phospho-epitopes), we next assessed the presence of more mature pathological markers like truncated tau (caspase 3 cleaved tau) and ThR-binding (filamentous tau). Caspase 3 can cleave tau at D421, a modification that occurs during the intermediate stages of tau tangle evolution in AD^68,69^. The TauC3 antibody, specific to this truncation site^55^, labeled relatively few tau inclusions in the treated neurons (Fig. 5A–D). Cleaved tau pathology (TauC3 antibody) primarily localized to neurites, but also some somatodendritic compartments of affected neurons, and did not typically colocalize with PAD-exposed tau (TNT1 antibody; Fig. 5A–D; fraction of TNT1 overlapping with TauC3 = 0.038 ±0.020). ThR labels β-pleated sheets of mature filamentous inclusions made up of tau or other amyloid-forming proteins^44,70–72^. We did not find evidence of filamentous tau structures (ThR positive) in any of the conditions (Fig. 5E-G). Primary delete stains confirmed there was no non-specific cross-reactivity between TNT1 and TauC3 or ThR (Supplementary Fig. 6). Collectively, these findings suggest most of the seeded pathology exists in the earlier phases of tau pathology evolution. However, a small subset of neurons developed more mature forms (e.g. cleaved tau pathology), but those are not yet adopting β-sheet structures associated with tau filaments that are formed in later stages of tangle evolution.

### Seeding MAPT-KI cultures does not result in overt degeneration

We determined that pathological tau did not induce overt toxicity in MAPT-KI cultures as measured by the CellTiter-Glo cell viability assay (Supplementary Fig. 7A) or the ApoTox-Glo assay for cell viability, cytotoxicity, or caspase-3/7 activity (Supplementary Fig. 7B-D). There were no cell type-specific effects as measured by immunoblotting culture lysates for neuronal β-III tubulin (Tuj1 antibody) and astrocytic GFAP (GFAP antibody; Supplementary Fig. 7E-G) protein levels or when the number of neurons (MAP2 antibody), astrocytes (GFAP antibody), or oligodendrocytes (MBP antibody; Supplementary Fig. 7H-M) were manually counted in PBS, Con and AD-tau treated cultures.

We assessed axonal degeneration in PBS, Con, and AD-tau neurons at DIV21 (Supplementary Fig. 8A–C) using two outcome measures: (1) the number of axonal objects/total axon area, and (2) the average axonal object size. A degenerated axon is expected to have a greater number of objects and a smaller average object size, due to fragmentation, when compared to an intact axon^73^. We observed no treatment effects for the number of objects/total axon area (Supplementary Fig. 8D) or for the mean object size (Supplementary Fig. 8E). Next, we measured calpain activity as an indirect measure of axonal degeneration^74–76^ but did not observe any treatment effects (Supplementary Fig. 8F). Finally, we used high-density MEA analysis to track action potential propagation along axonal arborizations of individual, treated neurons (Supplementary Fig. 8G-K). There were no treatment effects on total axon length, length of the longest branch (Supplementary Fig. 8I-J), or mean action potential conduction velocity (Supplementary Fig. 8K). Taken together, these results suggest that tau seeding does not cause overt cell death or axonal degeneration.

### Colocalization of active GSK3β with pathological tau inclusions

**Fig. 6:**
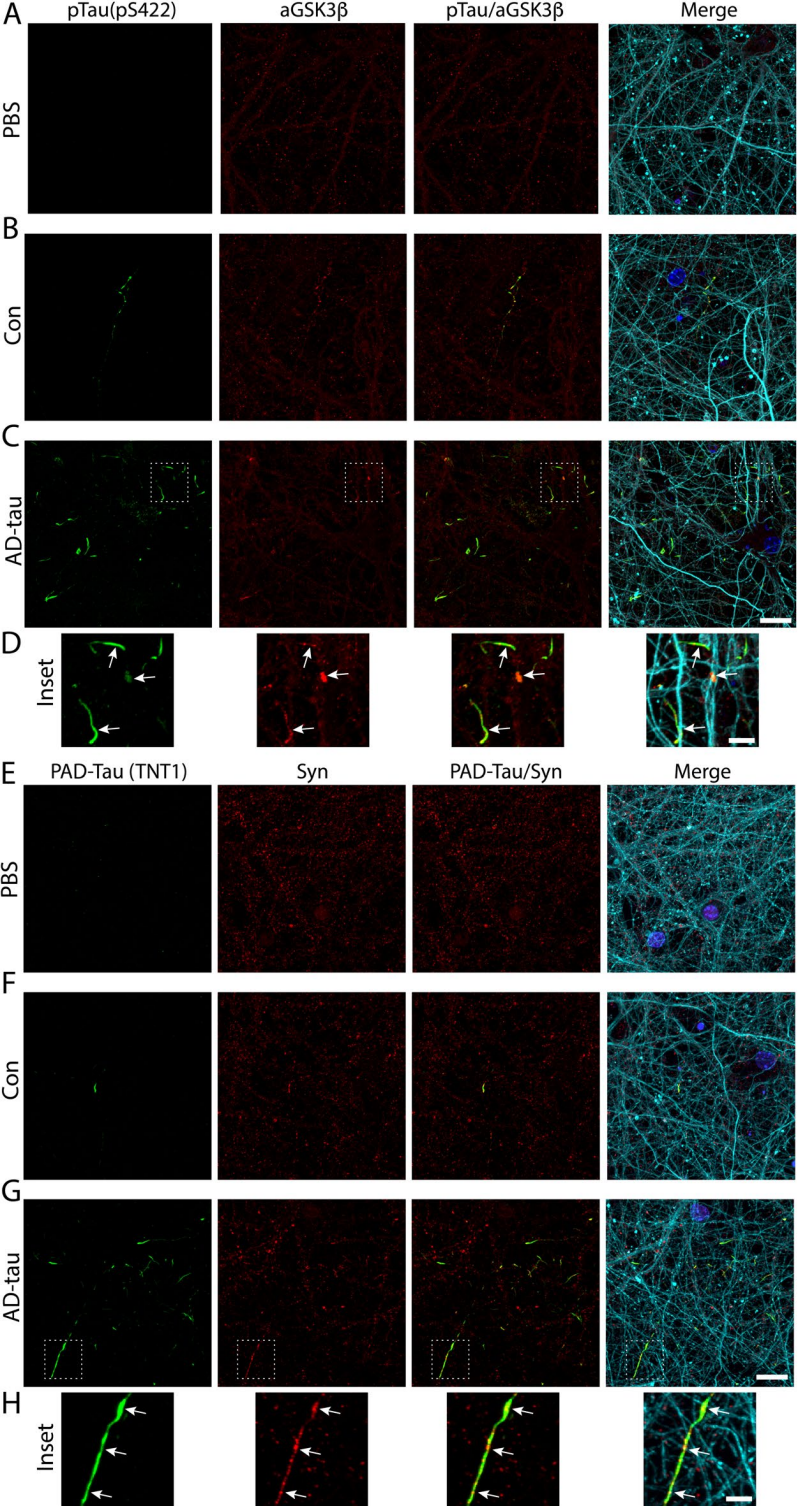
Active GSK3β and synaptophysin cargos co-accumulate with pathological tau inclusions. Cultures were treated on DIV5 with PBS, Con, or AD-tau and fixed at DIV21. A–C) Active GSK3β (aGSK3β; non-phospho-S9 GSK3β antibody; red) accumulations were found in some phospho-Ser422 tau inclusions (pTau; pS422 antibody; green) in AD-tau (**C**) and Con (**B**) treated neurons, but inclusions were not present in PBS treated cultures (**A**). As expected aGSK3β was present in all conditions (**A**–**C**). The merged images include pS422 pTau, aGSK3β, β-III tubulin (Tuj1 antibody; cyan) and a nuclear counterstain (DAPI; blue). Scale bars = 20 μm. (**D**) Insets show colocalization between pTau (pS422) and aGSK3β in some inclusions that appear as localized accumulations in the pTau (pSS22) inclusions (arrows). Scale bar = 5 μm. (**E**–**G**) Accumulations of synaptophysin (Syn; synaptophysin antibody; red) were found in PAD-exposed tau inclusions (PAD-Tau; green) in AD-tau (G) and Con (F) treated neurons, but inclusions were not present in PBS treated cultures (**E**). As expected, Syn was present in all conditions (**E**–**G**). Scale bars = 20 μm. (**H**) Insets show colocalization between PAD-tau and Syn, with some appearing as accumulations along the PAD-tau inclusions (arrows). Scale bar = 5 μm. *N* = 3.

Pathological exposure of PAD activates a protein phosphatase 1 (PP1)-GSK3β signaling cascade resulting in interruption of normal fast axonal transport^28–34^. To determine if there was aberrant PAD-pathway signaling in the AD-tau neurons, we immunostained PBS, Con, or AD-tau treated neurons for a marker of active GSK3β (aGSK3β; non-phospho-Ser9 GSK3β antibody;) and a marker of phospho-Ser422 tau (pS422 antibody) to identify pathological tau inclusions (Fig. 6A–D). We used the rabbit pS422 antibody as an alternate for PAD-exposed tau because TNT1 and aGSK3β antibodies are the same mouse isotype (note that pS422 extensively colocalized with TNT1 inclusions as shown in Fig. 4F-H). Accumulations of aGSK3β colocalized with some, but not all, pS422 phospho-tau inclusions (fraction of pS422 overlapping with aGSK3β = 0.328 ±0.065) in both the Con and AD-tau neurons (Fig. 6B-D). Primary delete stains confirmed there was no non-specific cross-reactivity between the pS422, β-III tubulin or aGSK3β antibodies (Supplementary Fig. 9A–D).

### Cargo proteins co-accumulate in discrete regions of neurites with PAD-exposed tau

Pathological tau-induced activation of GSK3β disrupts normal fast axonal transport^28–34,77,78^. To determine if cargo proteins accumulate along neurites in regions of PAD-exposed tau pathology, we performed ICF using the TNT1 antibody to detect PAD-exposed tau and synaptophysin (Fig. 6E-H) or amyloid precursor protein (APP; Supplementary Fig. 10A–C) antibodies to detect transport cargoes. Accumulations of synaptophysin signal frequently colocalized with the thread-like inclusions with PAD-exposed tau in Con and AD-tau neurons (Fig. 6F-H; fraction of TNT1 overlapping with synaptophysin = 0.483 ±0.034). We also observed APP-positive neurite swellings that were positive for PAD-exposed tau and β-III tubulin, resembling axonal spheroid bodies observed in AD tissue (Supplementary Fig. 10C). However, APP generally showed low colocalization with PAD-exposed tau inclusions (Supplementary Fig. 10B and C; fraction of TNT1 overlapping with APP = 0.152 ±0.041). Primary delete stains confirmed there was no non-specific cross-reactivity between the TNT1, Tuj1 and synaptophysin (Supplementary Fig. 9E-H) or APP antibodies (Supplementary Fig. 10D).

### AD-tau treatment significantly reduces the density of intact excitatory synapses

**Fig. 7:**
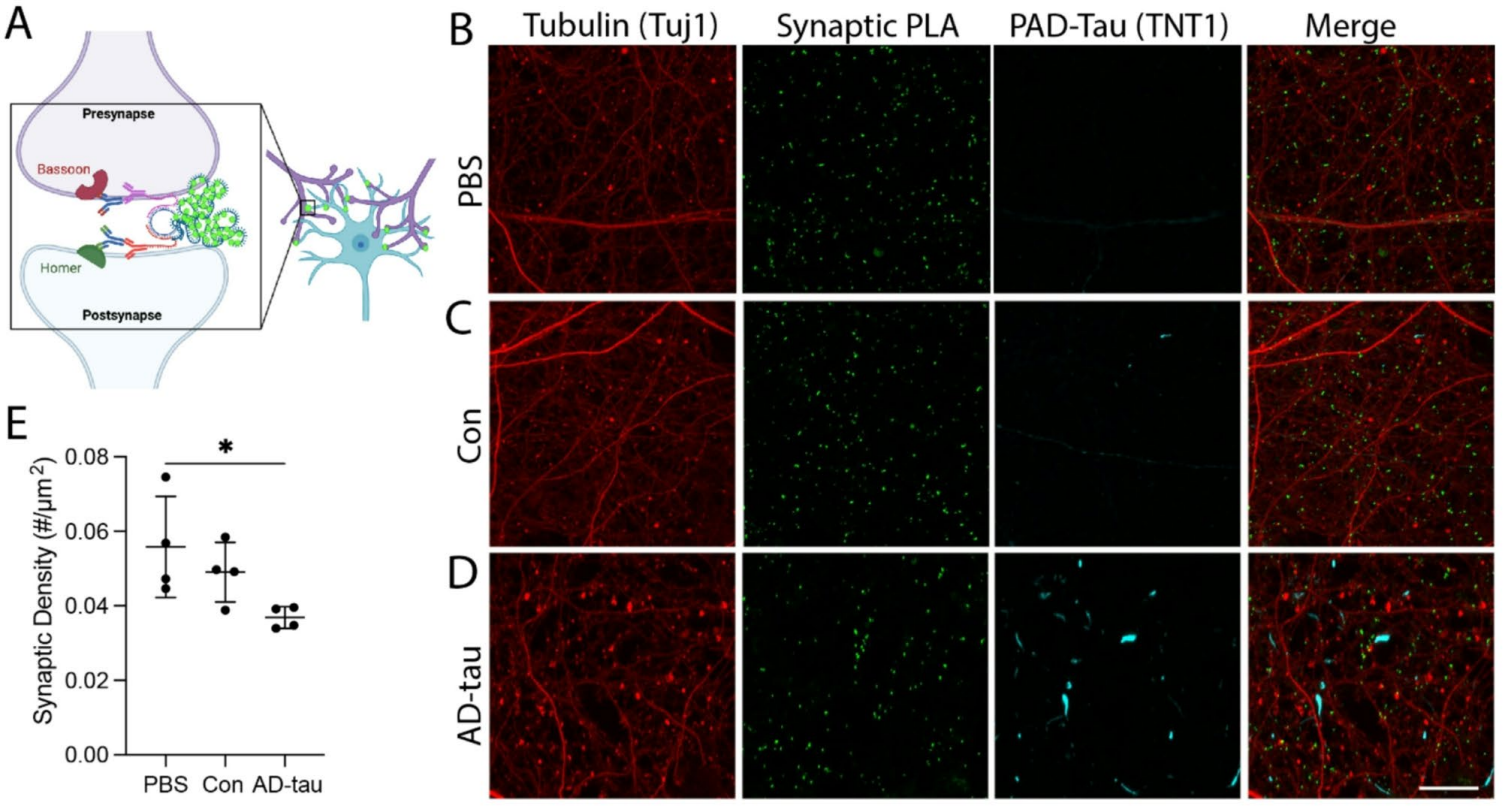
Excitatory synapse loss in MAPT-KI neurons treated with AD-tau. Cultures were treated with PBS, Con, or AD-tau at DIV5 and fixed on DIV33. (**A**) The proximity ligation assay (PLA) used homer 1 and bassoon antibodies to identify intact excitatory synapses (< 40 nm apart). Illustration generated using Biorender.com. (**B**–**D**) Representative images of homer-bassoon PLA labeling of intact excitatory synapses (Synaptic PLA; green puncta). After PLA, cells were immunolabelled for PAD-exposed tau (PAD-Tau; TNT1 antibody; cyan) and β-III tubulin (Tuj1 antibody; red). E) Intact excitatory synaptic density (expressed as the number of homer-bassoon PLA puncta per area of β-III tubulin staining) is significantly decreased in AD-tau cultures (~ 30% loss) when compared to PBS cultures. The data are mean ± SD and were compared using one-way ANOVA with Tukey’s multiple comparisons test, significance was defined as *p ≤ 0.05. Scale bar = 20 μm. *N* = 4.

Synaptic PLA can quantify intact excitatory synapses using the presynaptic bassoon protein and postsynaptic homer 1 protein because of the distance requirements for the reaction (< 40nM; Fig. 7A)^61^. We quantified the density of these synapses in PBS, Con and AD-tau treated primary hippocampal MAPT-KI cultures (Fig. 7B-E). Treatment with AD-tau significantly reduced the number of intact excitatory synapses (as measured by green PLA puncta) by ~ 30% when compared to the PBS control group (one-way ANOVA; *F*_(2,9)_ = 4.312, *p* = 0.0486; PBS vs. AD-tau *p* = 0.0424; Fig. 7F). Little to no non-specific PLA signal appeared with the primary delete controls in untreated cultures (Supplementary Fig. 11).

### AD-tau treatment causes neuron network hypersynchrony in MAPT-KI neurons

**Fig. 8:**
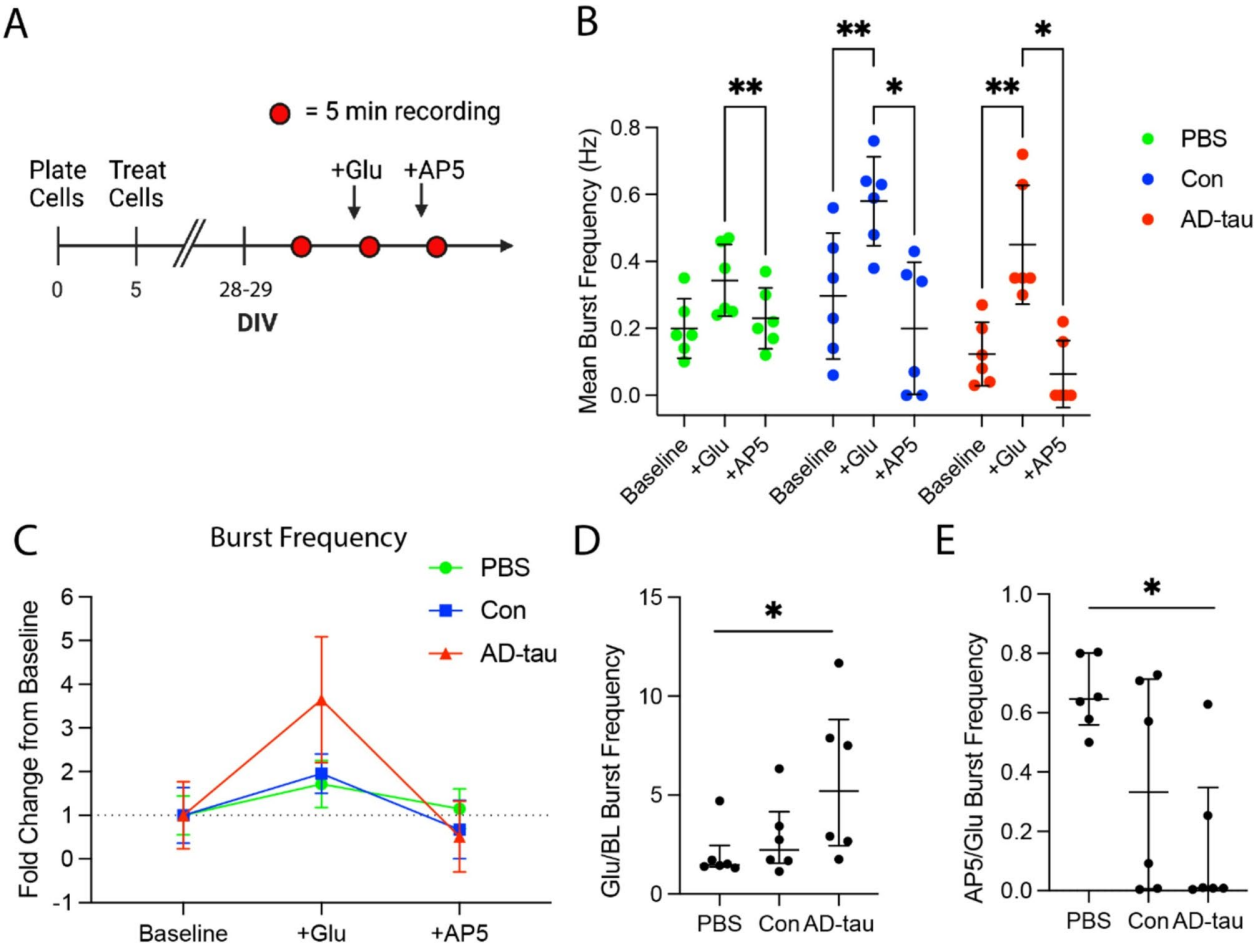
AD-tau treated cultures exhibit increased network hypersynchrony in response to glutamate. (**A**) Primary hippocampal MAPT-KI cultures were plated on high-density microelectrode array chips and treated with PBS, Con, or AD-tau on DIV5. On DIV28-29, three consecutive 5-minute neuronal activity recordings (red circles) were acquired. Basal network burst frequency was measured in cultures prior to any treatment (baseline, first 5-minute segment). After treatment with glutamate (+ Glu; 20 µM final concentration), the response to NMDAR activation was measured (second 5-minute segment). After the addition of NMDAR antagonist (+ AP5; 5 µM final concentration), the response to NMDAR inhibition was measured (third 5-minute segment). (**B**) Within groups, +Glu increased network burst frequency in PBS, Con and AD-tau treated cultures, but this reached statistical significance only in Con and AD-tau cultures. Within groups, +AP5 significantly reduced network bursts compared to + Glu in PBS, Con and AD-tau cultures. Comparisons between PBS, Con and AD-tau neurons showed there were no treatment effects on network burst frequency at baseline, after the addition of Glu, or after the addition of AP5. The data in B are mean ± SD and were compared using two-way repeated measures ANOVA with Tukey’s multiple comparisons test. (**C**) Within each group, the fold-change from mean baseline to + Glu and from mean baseline to + AP5 were calculated (data are mean ±SD). All three groups showed increased burst frequency in response to Glu, but the AD-tau cultures showed the greatest fold-change from their respective mean baseline values. All three groups showed reduced burst frequency following AP5 treatment, but the fold change was greatest in AD-tau cultures. (**D**–**E**) The magnitude of the response to Glu (+ Glu/Baseline; D) and then to AP5 (+ AP5/+Glu; **E**) was quantified. The AD-tau cultures exhibited a significantly greater change from baseline to + Glu (**D**), and from + Glu to + AP5 (**E**) compared to the PBS cultures. The data in D and E are median ± interquartile range and were compared using Kruskal-Wallis ANOVA with Dunn’s post hoc test. *p ≤ 0.05, **p ≤ 0.01. *N* = 6.

We measured the effect of tau seeding on neuronal function using high-density MEAs. We plated primary MAPT-KI neurons on MEA chips, treated on DIV5, then recorded activity at ~ 1,020 randomly selected electrodes over 5 min on DIV28-29 to establish a baseline activity in cultures. We then treated the cultures sequentially with the glutamatergic receptor agonist, Glu, followed by the NMDAR antagonist, AP5, and recorded neuronal activity at the same electrodes after the addition of each treatment for 5 min (Fig. 8A). This allowed us to calculate culture-wide changes in network hypersynchrony (i.e. network bursts) at baseline and in response to Glu or AP5 treatment.

First, we measured synchronous activity (i.e. periods of simultaneous firing followed by sparse activity, referred to as network bursts) to determine the effect of tau seeding on spontaneous and evoked network burst frequency in the cultures. Burst frequencies were measured at baseline and again following sequential Glu (expected to increase excitatory neuron activity) and AP5 (expected to reduce excitatory neuron activity) treatments in PBS, Con and AD-tau cultures using the recordings described above (Fig. 8B). Within the PBS cultures, Glu caused a 72% increase in neuronal network burst frequency compared to baseline, but this did not reach statistical significance (*p* = 0.059; Fig. 8B). Following AP5 treatment network burst frequency was significantly reduced compared to Glu-evoked network burst frequency in PBS cultures (Fig. 8B). In contrast, the addition of Glu significantly increased culture network burst frequency compared to baseline within Con (95% increase) or AD-tau (266% increase) cultures (Fig. 8B). Treatment with AP5 significantly reduced network burst frequency compared to Glu-evoked levels in PBS (33% reduction), Con (66% reduction) and AD-tau (86% reduction) cultures (Fig. 8B). Comparisons of network burst frequency across PBS, Con and AD-tau cultures did not show significant differences at baseline or after Glu and AP5 treatments (Fig. 8B; two-way repeated measures ANOVA; Recording (BL, +Glu, or + AP5) *F*_(2, 45)_ = 20.07 *p* = 0.0021; Treatment (PBS, Con, or AD-tau) *F*_(2, 45)_ = 3.476 *p* = 0.0921; Interaction *F*_(4, 45)_ = 3.260 *p* = 0.0937; PBS + Glu vs. +AP5 *p* = 0.0041; Con BL vs. +Glu *p* = 0.0037, +Glu vs. +AP5 *p* = 0.0252; AD-tau BL vs. +Glu *p* = 0.0068, +Glu vs. +AP5 *p* = 0.0111). To illustrate the relative response of each treatment group (PBS, Con, AD-tau) to Glu and AP5, we plotted the data as fold-change from mean baseline of each group (Fig. 8C). All three groups showed increased burst frequency from baseline in response to Glu (PBS = 1.72-fold; Con = 1.96-fold; AD-tau = 3.65-fold) and similar or reduced burst frequency from baseline in response to AP5 (PBS = 1.15-fold; Con = 0.67-fold; AD-tau = 0.51-fold). Notably, the AD-tau cultures showed the largest response to Glu and AP5 relative to PBS and Con cultures.

In response to the variability in network burst frequency within and across groups, we normalized the values in response to Glu treatment to the pre-treatment baseline values and the values in response to AP5 treatment to the Glu-evoked values for each independent replicate. The magnitude of the Glu-evoked response (change from baseline to + Glu) was significantly greater in the AD-tau cultures compared to PBS (Fig. 8D; Kruskal-Wallis ANOVA with Dunn’s post hoc, H = 7.638, *p* = 0.0150; PBS vs. AD-tau *p* = 0.0172). The magnitude of the AP5-evoked response (change from + Glu to + AP5) was also significantly greater in the AD-tau cultures compared to PBS cultures (Fig. 8E; Kruskal-Wallis ANOVA with Dunn’s post hoc, H = 6.334, *p* = 0.0350; PBS vs. AD-tau *p* = 0.0385).

We also measured mean action potential firing rate using the same treatment paradigm (Supplemental Fig. 12A). Within group comparisons showed a non-significant elevation in mean firing rate within PBS cultures treated with Glu (77% increase, *p* = 0.13) when compared to baseline and a significant decrease in mean firing rate with AP5 (21% decrease) compared to Glu (Supplementary Fig. 12B, C). Within Con and AD-tau cultures there was a significant 54% and 105% increase in mean firing rate with Glu when compared to baseline, respectively. AP5 caused a significant reduction in mean firing rate within Con (43% decrease) and AD-tau (68% decrease) when compared to Glu (Supplementary Fig. 12B). We found no significant differences in mean firing rate after Glu or AP5 treatments when comparing between PBS, Con, or AD-tau conditions (Supplementary Fig. 12C-E) although a trend was observed in which AD-tau cultures showed stronger inhibition with AP5 treatment when compared to PBS cultures (Supplementary Fig. 12E; *p* = 0.06).

To validate that tau seeding occurred in the MEA cultures, we collected the cells after the final recording and performed sELISAs for total tau (Tau5 antibody), PAD-exposed tau (TNT1 antibody), and oligomeric tau (TOC1 antibody) (Supplementary Fig. 13). Total tau was slightly increased in the AD-tau treated cultures compared to Con treated cultures (Supplementary Fig. 13A and B). AD-tau cultures had significantly higher levels of PAD-exposed tau (Supplementary Fig. 13C and D) and oligomeric tau (Supplementary Fig. 13E and F) when compared to PBS and Con treated cultures, which had little to no detectable PAD-exposed or oligomeric tau.

## Discussion

We developed a model of tau seeding in MAPT-KI primary hippocampal cultures by treating cultures with human AD brain-derived seeding material. Intracellular tau inclusions in this model required recruitment of endogenous tau and displayed several conformational and post-translational modifications associated with early tau deposition in human AD but did not progress to mature filamentous inclusions within four weeks. These pathological changes are linked to neuronal dysfunction and degeneration in disease. The tau inclusions primarily localized to the axons of neurons, although we identified occasional somatodendritic inclusions as well. Affected neurons displayed signs of axonal dysfunction, abnormal synaptic function and synaptic loss associated with the formation of tau inclusions but we did not detect overt degeneration.

We characterized the maturity of the tau inclusions using several well-characterized pathological tau markers. These included phospho-tau antibodies, AT8, pS422 and PHF1, that are used extensively in the AD field to identify pathological tau, appearing in early stages of disease and persisting through late stages. We also used well-characterized conformation-specific antibodies that detect exposure of an N-terminal domain known as PAD (TNT1) and tau oligomers (TOC1), both of which appear early in human disease^29,52,79^. Several of these pathological tau markers colocalize in human disease. For example, our group found that AT8 colocalizes with PAD-exposed tau in brain tissue from AD^29,79^. Molecular studies showed that pseudophosphorylation at the AT8 sites in recombinant tau results in exposure of PAD^80^. Furthermore, pS422 tau and tau oligomers (TOC1) also colocalize with PAD-exposed tau in human tauopathy brains^34,67^. Consistent with this, the tau inclusions observed in our primary neuron seeding model showed colocalization between PAD-exposed tau, AT8, pS422 and oligomeric tau. The conformational and post-translational modifications labelled by these markers precede tau filament formation. Caspase cleaved tau (TauC3-positive, truncated at D421) typically occurs during the intermediate to late stages of tangle formation^68,81^, and mature tau filaments contain β-sheet structures identifiable using ThR. We identified relatively few cleaved tau inclusions, which typically did not colocalize with PAD-exposed tau, and we found no evidence of filamentous tau inclusions. Together, these data indicate that the tau inclusions formed in the present seeding model have characteristics of pretangle tau pathology.

Notably, the tau inclusions were primarily located in the axons, with rare somatodendritic localization. This is consistent with a report from Guo and colleagues showing tau seeding is largely restricted to the axonal compartment in wildtype mice treated with AD-tau^5^. Our group established that oligomeric (TOC1+)^77^ and phosphorylated (AT8 + and pS422+)^29,30,34^ forms of tau are mechanistically linked to axon transport disruption through a PAD-PP1-GSK3β signaling pathway that ultimately causes cargo release from kinesin motor proteins and impaired fast axonal transport^29,33,78^. Disruption to normal axonal transport can lead to a number of deleterious effects in neurons, including synapse dysfunction, synaptic loss, and axonal degeneration.

We observed signs of axonal transport impairment in the seeding model that were localized to sites of the tau inclusions. Accumulations of active GSK3β signal colocalized with some pS422-positive tau inclusions, suggesting “hot-spots” of GSK3β activity at the tau inclusions (Fig. 6). Axonal GSK3β activity can cause cargo release from motor proteins, which may explain the accumulation of synaptophysin and some APP at PAD-exposed tau inclusions. This is consistent with a recent study by our group showing that expression of monomeric AT8 phosphomimetic tau in rat primary neurons significantly increased the pausing frequency of axonal synaptophysin-positive cargoes^30^. Recently, another group reported tau accumulation in distal axons and a significant reduction in the pool of moving lysosomes along axons in primary hippocampal neurons transfected with a tau phosphomimetic^82^. PAD-exposure was not assessed in these studies, though the findings support a model of increased cargo pausing, similar to our observations in WT rat neurons transfected with AT8-tau or with FTD-mutant tau^30,31^. Additionally, dystrophic axons containing PAD-exposed tau in our model were reminiscent of axonal dystrophy and spheroid bodies in post-mortem early AD tissue^18,20^ and in animal models of tauopathy^25–27,83^. Spheroids and dystrophic neurites are thought to result from axonal transport dysfunction^84–86^. Together, these findings provide indirect evidence of axonal transport dysregulation due to discrete regions of aberrant PAD-exposure and GSK3β activation.

Despite signs of axonal transport dysregulation, we did not observe overt axonal degeneration. Axonal abnormalities and degeneration are common features of multiple tauopathy animal models^24–27,83,87^ but evidence of axonal degeneration in tau seeding studies is sparse. Clavaguera, et al., showed no overt axonal degeneration despite extensive tau pathology in human 2N4R tau expressing mice injected with brain homogenate from mice expressing human 0N4R P301S mutant tau^88^. However, some evidence for delayed axonal degeneration exists in other models using spatially-restricted expression of P301L^21^, phosphomimetic forms of tau^89^, or injection with human AD-tau^90^ to model tau seeding. It is possible that we did not observe overt axonal degeneration in our seeding model because the 28d post-treatment timeframe is too short. The presence of early axonopathy in this model, including axonal swellings and the accumulation of cargos and active GSK3β, suggests an impairment of transport that may precede eventual axonal degeneration. Pathological tau formed in only a subset of axons which presented technical challenges for detecting degeneration in culture-wide assays or identifying degeneration and transport disruptions in specific axons containing inclusions.

Synapse dysfunction and loss are early events in the pathogenesis of AD and are logical potential consequences of axonal transport impairment^13–16,91–93^. We observed a significant loss (~ 30%) of intact excitatory synapses in AD-tau treated neurons compared to PBS controls. These findings are consistent with reports of a loss of presynaptic and postsynaptic markers in tauopathy models^21–23,94–96^. Most of these previous studies used models of tau overexpression, often with mutations associated with inherited tauopathies, while tau seeding studies using insoluble AD-tau did not assess synaptic density^5,10,12,98^, with the exception of one study that reported reduced synaptophysin and PSD95 levels in WT mice injected with AD-tau^99^. The changes in synaptic proteins occurred in the absence of overt neuron loss, which aligns with modeling early effects in response to tau seeding. The lack of overt cell toxicity (as measured by multiple assays) in our model is consistent with the majority of *in vitro* and *in vivo* tau seeding studies^5,10–12,100^.

We also observed evidence of synaptic dysfunction in the AD-tau neurons, specifically NMDAR-dependent, glutamate-induced culture-wide network hypersynchrony. Hypersynchrony is a neuronal phenotype associated with AD^101^. Functional magnetic resonance imaging (fMRI) studies in humans show hyperactivity in the hippocampus during memory-related tasks at the early stages of MCI^35,36^. Interestingly, hypoactivity is observed in later stage MCI and in AD, suggesting that hyperactivity is transient and an early physiological change in the brain of individuals at risk for developing AD^35,36^. Similarly, neuronal hypersynchrony is observed in models of AD^102,103^ and can manifest as clinical or subclinical epilepsy, which can occur during the early stages of AD^104–110^. The increased hypersynchrony in our AD-tau cultures suggests that seeded tau inclusions in a subset of neurons are sufficient to alter normal network activity. Hyperexcitability of individual neurons is another AD-linked phenotype, but we were unable to detect significant changes at the electrode level, again, likely due to the challenges of assigning effects to individual neurons with or without inclusions.

The use of MAPT-KI mice provides several advantages for our model system. The knock-in of human *MAPT* ensures endogenous levels of WT tau expression. This is important for studying tau-mediated effects in sporadic AD, which accounts for over 95% of AD cases and is not associated with mutant tau or tau overexpression^111^. Additionally, adult MAPT-KI mice express all 6 major tau isoforms found in the adult human brain. Adult WT mice express only 4R tau isoforms and the primary amino acid sequence of mouse tau differs from human tau (especially in the N-terminus). Paired helical filaments and straight filaments in human AD tissue are comprised of both 3R and 4R tau isoforms so having 3R and 4R tau isoforms expressed in the model is advantageous for recapitulating that aspect of disease. The tau isoform expression profile in MAPT-KI primary neurons was previously unknown, and here we show that by DIV14, the neurons express both 3R and 4R tau isoforms. Furthermore, the MAPT-KI cultures include a mix of neurons and astrocytes with a minor oligodendrocyte component (~ 1%). Due to the important role of astrocytes at the synapse, their presence may be especially important for studies of synaptic function.

## Conclusions

We found that adding insoluble tau purified from human AD brains to primary neurons expressing physiological levels of 3R and 4R human tau isoforms results in the formation of tau pathology. The pathology displays several markers of early tau dysfunction including conformation changes, the formation of tau oligomers, and phosphorylation at disease-associated sites. The seeded pathologies were associated with axonal dystrophy and synaptic dysfunction and loss, but without overt neuron degeneration. This supports a model whereby pathological forms of pretangle tau can induce inceptive cellular dysfunction. Indeed, this model appears well-positioned to serve as a tool to understand how pretangle tau pathology affects synaptic and axonal function and how additional stressors may contribute to the development of overt neurodegeneration. From a novel therapeutic development perspective, it may prove useful in testing inhibitors of tau aggregation, spreading and early mechanisms of tau-mediated toxicity.

## Supplementary Information

Below is the link to the electronic supplementary material.

Supplementary Material 1

## Data Availability

Data is provided within the manuscript or supplementary information included.
