# Supplementary material for: Seeding Alzheimer’s disease-associated tau pathology in MAPT knock-in primary neurons causes early axonopathy and synaptic dysfunction
Source: Sci Rep. 2025 Oct 31;15:38140. doi: 10.1038/s41598-025-21920-8 (PMC12578843; doi:10.1038/s41598-025-21920-8)
Supplement: Supplementary file 1 — Supplementary Material 1 [file 41598_2025_21920_MOESM1_ESM.docx]

**Supplementary Material**

**Seeding Alzheimer’s disease-associated tau pathology in *MAPT* knock-in primary neurons causes early axonopathy and synaptic dysfunction**

Rebecca L. Mueller, Benjamin Combs, and Nicholas M. Kanaan

**Supplementary Methods**

*Transmission electron microscopy (TEM)*

TEM was used to visualize the tau aggregates in the AD-tau seeding material. Samples were incubated on 300 mesh Formvar carbon coated copper grids (Electron Microscopy Sciences, 215-412-8400) for 1 min. Grids were washed 1x with ultrapure water, 1x with 2% uranyl acetate, and negatively stained with 2% uranyl acetate for 1 min. Grids were imaged using a JEOL JEM-1400 Plus electron microscope at 80 kV equipped with an AMT XR81 digital camera and AMT software version 602.6 (Advanced Microscopy Techniques).

*Western blotting for seeding material quality control*

Immunoblotting for 4R and 3R isoforms and phosphorylated tau was performed to characterize the tau present in the brain-derived seeding material. Total protein concentrations of Con and AD-tau samples were quantified using the SDS-Lowry protein quantitation assay (see below). Samples were prepared for SDS-PAGE (2 µg/lane), separated on 10% Criterion TGX Precast Midi Protein Gels (Bio-Rad, 5671034) for 32 min and transferred to nitrocellulose as described in the manuscript Methods section. One membrane was probed to detect 4R tau isoforms (4R antibody; 1:2,000; rabbit polyclonal; CosmoBio, TIP-4RT-P01; AB_2814647) and 3R tau isoforms (3R antibody; 1:1,000; mouse IgG1; Millipore, 05-803; AB_310013). A second membrane was probed for phospho-S396/S404 tau (PHF1 antibody) and total tau (R1 antibody; 1:100,000; rabbit polyclonal). Membranes were incubated with IRDye 680LT goat anti-mouse IgG1 and IRDye 800CW goat anti-rabbit. Membranes were imaged using a Licor Odyssey infrared imaging system with Licor ImageStudioLite 5.2 software.

*Sandwich enzyme-linked immunosorbent assays (sELISAs) with human derived AD-tau and Con samples*

To measure total tau and the presence of pathogenic tau conformations in the AD-tau and Con samples, non-denaturing sELISAs were used as described in the manuscript Methods section. The capture antibodies used were for total tau (Tau5 antibody), PAD-exposed tau (TNT1 antibody), or oligomeric tau (TOC1 antibody). For total tau quantification, standard curves using 2N4R tau monomers (prepared as previously described [1]) were serially diluted in TBS (80 nM – 0.0625 nM; 1:2 dilution). AD-tau and Con samples were diluted 1:2500 and 1:5000, and additional Con samples were prepared at 1:250 and 1:500 in TBS. For identification of pathological tau species, 2N4R tau monomers and aggregates were serially diluted 1:4 from 80 nM to 0.001 nM (Tau5 and TNT1 assays) or from 200 nM – 0.003 nM (TOC1 assays). A rabbit polyclonal total tau antibody (R1 antibody; 50 µl/well; 1:5,000) was used as the detection antibody followed by goat anti-rabbit antibody conjugated to horseradish peroxidase diluted 1:5,000. Signal was detected by adding 50 µl/well of 3,3’,5,5’ tetramethylbenzidine substrate (Sigma, T0440). The reactions were quenched with 50 µl/well of 3.6% H_2_SO_4_ and absorbance was read at 450 nm. Absorbance data were converted to percent light absorbed as described in the manuscript Methods section.

*SDS-Lowry Protein Quantitation Assay*

To calculate the percentage of tau protein in the AD-tau and Con samples in relation to total protein, we used the SDS-Lowry method to quantify total protein as previously described [1]. Briefly, bovine serum albumin (BSA; Thermo Fisher Scientific, 23209) protein standards, AD-tau and Con samples were diluted in SDS solution (2% SDS/5% 2-mercaptoethanol/10% glycerol in 0.0625M Tris, pH 6.8) to a final volume of 100 µl. Next, 1 mL of 10% perchloric acid/1% phosphotungstic acid solution was added to each sample. After incubating on ice for 1 h, samples were centrifuged at 18,000 x g for 15 min at 4 °C. The supernatant was discarded, and the pellets were dried. Each pellet was dissolved in Lowry solution (0.01% CuSO_4_, 0.02% Na_2_CO_3,_ 0.001N NaOH) and incubated for 10 min at room temperature. Finally, 100 µl of 1X Folin-Ciocalteu’s Phenol Reagent (Sigma-Aldrich, F9252) was added to each sample and samples were incubated for 45 minutes. Absorbance values were read at 750 nm with a spectrophotometer, and the total protein concentrations were interpolated from the BSA standard curve.

*Tau RD P301S cell seeding assay*

The RD Biosensor Cell line (Tau RD P301S FRET Biosensor cells; ATCC, CRL-3275) was used to confirm whether the AD-tau samples were seed competent. Cells were plated in a poly-D-lysine-coated 96-well plate (Corning, 354461) density of 12,000 cells/well in 100 µl/well of RD media [DMEM (Gibco, 11995-065), 10% FBS, 1% Pen/Strep (Gibco, 15140-122), 1X GlutaMAX (Gibco, 35050-061)]. The next day, cultures were treated with PBS, Con or AD-tau delivered via lipofection. Each treatment (PBS, Con or AD-tau) was diluted in OptiMEM (Gibco, 31985-062) to 28 nM AD-tau and Con matched to AD-tau for total protein amount. In parallel, Lipofectamine 2000 (Thermo Fisher Scientific, 11668019) was diluted in OptiMEM. After 20 min, the treatment and Lipofectamine reagents were combined for an additional 20 min. Next, the Lipo/Treatment mixtures were added to the cells (20 µl/well). After 48 hours, cells were fixed with 37 °C pre-warmed 4% paraformaldehyde in fixation buffer for 20 min, then washed 3 x 5 min with TBS. DAPI (0.5 µg/mL) was added to the first wash. Cells were imaged using a Nikon Eclipse Ti2 Inverted microscope equipped with a Zyla sCOMS camera (Andor), a S Plan Flour 20x (0.45 numerical aperture) objective, DAPI filter (excitation 383-408 nm, emission 435-485 nm), GFP filter (excitation 450-490 nm, emission 500-550 nm), TexasRed filter (excitation 540-580 nm, emission 593-668 nm), and NIS Elements software (Nikon, v5.02.00).

*Immunocytofluorescence of PBS, Con, or AD-tau treated primary MAPT-KI and Tau-KO cultures*

Immunostaining was used to show that tau seeding does not occur in AD-tau treated Tau-KO neurons and to assess potential tau seeding in astrocytes. Furthermore, the number of neurons, astrocytes and oligodendrocytes were manually counted to determine cell-specific overt toxicity. The following combinations of antibodies were used for ICF as described in main Methods:

1) PAD-exposed tau (TNT1 antibody; 1:20,000) + β-III tubulin (Tuj1 antibody; 1:5,000); Alexa Fluor goat anti-mouse IgG1 568 + Alexa Fluor goat anti-mouse IgG2a 488.

2) PAD-exposed tau (TNT1 antibody; 1:40,000) + an astrocyte marker (GFAP antibody; 1:4,000); Alexa Fluor goat anti-mouse IgG1 568 + Alexa Fluor goat anti-rabbit 488.

3) Microtubule associated protein 2 (MAP2 antibody; 1:10,000) + an astrocyte marker (GFAP antibody; 1:4,000); Alexa Fluor goat anti-mouse IgG1 488 + Alexa Fluor goat anti-rabbit 568.

4) MBP (1:2,000); Alexa Fluor goat anti-chicken 488.

5) PAD-exposed tau (TNT1 antibody; 1:40,000) + β-III tubulin (Tuj1 antibody; 1:5,000) + amyloid precursor protein (APP antibody; 1:100; rabbit; Abcam, ab32136; AB_2289606); Alexa Fluor goat anti-mouse IgG1 488 + Alexa Fluor goat anti-mouse IgG2a 647 + Alexa Fluor goat anti-rabbit 568.

For the primary delete experiments, the stains were performed at the same time as the stains shown in the main paper (the same antibody solutions were used), and therefore the methods for these are the same as written in the main Methods section. Images were acquired with confocal microscopy as described in the main Methods section. For the cell-type specific toxicity ICF stains, images were acquired with a Nikon Eclipse Ti2 Inverted microscope (as above).

*Overt Cell Toxicity Assays*

To determine if the pathological tau in the MAPT-KI cultures was toxic to the cells, two cell viability assays were used. First, the CellTiter-Glo Assay kit (Promega, G7570) was used to measure cell viability via quantifying ATP levels, which is proportional to the number of viable cells. Cells were plated in a poly-D-lysine coated 96-well plate (Corning, 354461) at 25,000 cells/well in 100 µl/well of NBM+. At 28d post-treatment, ATP was quantified using a CellTiter-Glo Assay kit according to the manufacturer’s instructions. CellTiter-Glo reagent was added directly into the cell media at a 1:1 ratio. The reaction was mixed on a shaker at room temperature for 2 min, then developed for 10 min. Then, each reaction was transferred to a solid white well plate (Corning, 3912) and luminescence was recorded using a Promega GloMax plate reader.

The second assay used was the ApoTox-Glo Triplex assay (Promega, G6320) that measures cell viability, cytotoxicity and caspase-3/7 activity. Cells were plated in a black walled/clear bottom poly-D-lysine coated 96-well plate (Thermo Fisher Scientific, 152037) at 25,000 cells/well in 100 µl/well of NBM+. At 26d post-treatment, cell viability, cytotoxicity and caspase activity were measured using an ApoTox-Glo Triplex Assay kit as directed. First, 20 µl of Viability/Cytotoxicity Reagent was added directly to each well and the reaction was mixed on a shaker at room temperature for 30 s, then returned to incubator for 1 h. Fluorescence was measured using a Promega GloMax plate reader with the following filter cubes: 405_Ex_/495-505_Em_ for viability; 490_Ex_/510-570_Em_ for cytotoxicity. Then, 100 µl of Caspase-Glo 3/7 Reagent was added to each well and the plate was mixed for 30 s then incubated at room temperature for 30 min. Then, 180 µl of each reaction was transferred to a solid white well plate and luminescence was recorded using a Promega GloMax plate reader. At each time point indicated, samples from each experimental group were run together in the assay.

*Immunoblotting of neuronal and astrocytic proteins*

Immunoblotting was used to measure the level of neuronal and astrocyte protein expression. Samples (15 µg/lane) from 26d post-treatment (DIV31) cultures were processed, separated on Criterion TGX Precast 4-20% gels (Bio-Rad, 567-1095) via SDS-PAGE for 32 min and transferred to nitrocellulose as described in the manuscript Methods section. The membrane was cut just above the 37 kDa marker. The top portion of the membrane was probed for β-III tubulin (Tuj1 antibody; 1:10,000) and an astrocyte marker (GFAP antibody; 1:1,000), while the bottom portion of the membrane was probed for a protein loading control (GAPDH antibody; 1:2,000; rabbit; Cell Signaling Technology, 5174). The top portion of the membrane was incubated with IRDye 800CW goat anti-mouse IgG2a and IRDye 680LT goat anti-rabbit (Li-Cor Biosciences, 926-68021) and the bottom portion of the membrane was incubated with IRDye 680LT goat anti-rabbit. Membranes imaged using a Li-Cor Odyssey infrared imaging system. Li-Cor ImageStudioLite 5.2 Software was used to quantify the signal intensity of the bands. Signal intensities for the neuronal marker (Tuj1) and astrocyte marker (GFAP) bands were normalized to the loading control (GAPDH) bands.

*Axonal degeneration measurements*

Confocal microscopy image analysis was used to determine whether treated neurons showed signs of overt axonal degeneration. Glass bottom chamber slides (8-well; Ibidi, 80827) were coated with 0.5 mg/mL poly-D-lysine in borate buffer overnight at room temperature. Slides were washed 4x with sterile water and air dried. Slides were warmed prior to plating. Cells were plated as two micro-islands/well (1 island = 10 µl drop of 15,000 cells/drop) arranged diagonally. Cells were allowed to settle for 12 min in the incubator. Then, the volume was raised to 250 µl/well with NBM+. Cultures were treated at DIV5 with PBS, CO, or AD-tau. On DIV21, cells were fixed with prewarmed 4% paraformaldehyde in fixation buffer. Then, using ICF (as described in the manuscript Methods) cells were stained with markers for the neuronal somatodendritic compartment (MAP2 antibody; 1:250; Cell Signaling, 8707S), β-III tubulin (Tuj1 antibody; 1:5,000), and PAD-exposed tau (TNT1 antibody; 1:40,000). The Alexa Fluor goat anti-mouse IgG1 647, Alexa Fluor goat anti-mouse IgG2a 568, and Alexa Fluor goat anti-rabbit 488 secondary antibodies were used.

Cells were imaged at 20x with a Nikon A1+ laser scanning confocal microscope equipped with 405, 488, 561, and 640 solid state lasers and Nikon Elements AR software. For each condition, an entire micro-island was imaged by acquiring and stitching multiple fields of view using the Nikon Elements AR software. Images were analyzed using FIJI ImageJ software (version 2.14.0/1.54f). First, the channels were split using the Split Channel function. Next, a threshold mask was generated for the β-III tubulin signal (Tuj1 antibody; red channel) using the Huang setting. Next, the poly line tool was used to outline the somatodendritic compartment (MAP2 antibody reactive area) of each island, then the MAP2-positive area was deleted so the subsequent analysis was performed on axons (i.e., Tuj1-positive/MAP2-negative processes). Using the polyline tool, we traced the outer perimeter of the axon growth area. Next, the number of axonal segments were quantified using the Analyze Particles function (Size: 1-Infinity, Circularity 0.00 – 1.00).

*Axon tracking using high-density MEAs*

MAPT-KI primary neurons were plated on MEAs (as described in the manuscript Methods), treated on DIV5, and recorded 23d post-treatment (DIV28). All cultures were fed 80 µl of fresh NBME 1 h prior to the first activity recording. The chip was placed into the recording unit and allowed to equilibrate for 10 min. Then, an ActivityScan Assay was performed using a checkerboard configuration with the time per configuration set to 30 sec. Immediately following the ActivityScan, an AxonTracking Assay was performed. Sixty units (neurons; 9 electrodes per unit) with a minimum spacing of 175 µm were selected for recording. The selection preference was set to firing rate with no minimum spike amplitude. The density for scanning electrodes was set to Full, and the scanning mode was set to Full Array, Record time was set to 60 sec, time window before Action Potential was 1.5 ms, and time after Action Potential was 5.0 ms. The activity was recorded (approximately 1 h and 30 min) and then the AxonTracking Analysis was run. For the analysis, the number of spikes threshold was set to 20, the footprint completeness threshold was set to 0.75, the option to plot branch level selected, the latency threshold was set to 0 ms, and the radius was set to 3 pixels.

*Calpain-Glo Assay*

The level of calpain activity in treated cultures was measured at 26d post-treatment (DIV31). Cultures were plated in a PDL-coated 96-well plate (Corning, 354461) at 25,000 cells/well in 100 µl/well of NBM+. Calpain activity was measured using a Calpain-Glo Assay kit (Promega, G8501) according to the manufacturer’s instructions. Calpain-Glo reagent was added directly into the cell media at a 1:1 ratio. The reaction was mixed on a shaker at room temperature for 30 s, then developed for 30 min. Then, 50 µl each reaction was transferred to a solid white plate and luminescence was recorded using a Promega GloMax plate reader.

*Glutamate- and NMDA receptor antagonist evoked neuronal activity using MEAs*

The mean firing rate data were obtained from the same recordings used for the network analyses (see manuscript Methods section). An Activity Analysis was performed on each recording with a firing rate threshold of 0.1 Hz, an amplitude threshold of 20 µV, and an interspike interval threshold of 200 ms. We excluded data from electrodes with a spike amplitude of < 100 µV. Data from the Activity Analysis were handled three ways. First, mean firing rate values (Hz) are reported and used for statistical comparison. Second, mean firing rate data were normalized to the mean baseline values within each group to illustrate change from baseline in response Glu or AP5 treatment. Third, electrode-level firing rate data following Glu treatment were normalized to baselines for each independent replicate, and data following AP5 treatment were normalized to Glu values for each independent replicate and used for statistical comparisons.

*sELISAs of PBS, Con, and AD-tau treated culture lysates collected from MEAs*

To confirm the presence of pathological tau in the MEA experiments, cell lysates from PBS, Con and AD-tau treated MEAs were collected after the last recording and sELISAs were conducted as above. The cells were collected in 100 µl of cell lysis buffer (20 mM Tris, 0.5 mM dithiothreitol, 150 mM NaCl, 0.5% Triton X-100, 2 µg/mL pepstatin, 2 µg/mL bestatin, 2 µg/mL leupeptin, 4 mM phenylmethylsulfonyl fluoride, 10 µg/mL aprotinin, 1 mM tetra-sodium pyrophosphate decahydrate, 10 mM β-glycerophosphate, 1 mM sodium orthovanadate, 1 M sodium fluoride, pH 7.5). Lysates were sonicated and protein concentration quantified using the Bio-Rad protein assay as directed. The sELISA plates were coated with antibodies to capture total tau (Tau5 antibody), oligomeric tau (TOC1 antibody) and PAD-exposed tau (TNT1 antibody) as above. Recombinant 2N4R tau monomer and aggregate standard curves were prepared by diluting 80 nM 1:3 to 0.137 nM (Tau5 assay) or diluting 200 nM 1:3 to 0.137 nM (TNT1 and TOC1 assays). Lysates were adjusted to 5 µg (Tau5 assay) or 20 µg (TNT1 and TOC1 assays) in TBS. sELISAs were performed as above with a rabbit polyclonal pan tau detection antibody (R1 antibody; 1:10,000).

**Supplementary Tables**

**Table S1. Colocalization threshold setting in Just Another Colocalization Plugin analysis.**

| **Antibody Stain (Channel)** | **Threshold Channel A** | **Threshold Channel B** |
| --- | --- | --- |
| TNT1 (A) + TOC1 (B) | 241-248 | 1001-1010 |
| TNT1 (A) + AT8 (B) | 167-175 | 208-228 |
| TNT1 (A) + pS422 (B) | 246-249 | 182-183 |
| TNT1 (A) + PHF1 (B) | 246-249 | 292-294 |
| TNT1 (A) + TauC3 (B) | 195-218 | 199-212 |
| pS422 (A) + aGSK3β (B) | 563-570 | 390-400 |
| TNT1 (A) + Synaptophysin (B) | 238-246 | 291-304 |
| TNT1 (A) + APP (B) | 250-251 | 273-293 |

TNT1 – PAD-exposed Tau; TOC1 – oligomeric tau; AT8 – phospho-tau; pS422 – phospho-tau; PHF1 – phospho-tau; TauC3 – cleaved tau; aGSK3β - active GSK3β (non-phospho-S9 GSK3β); APP – amyloid precursor protein.

**Supplementary Figures**


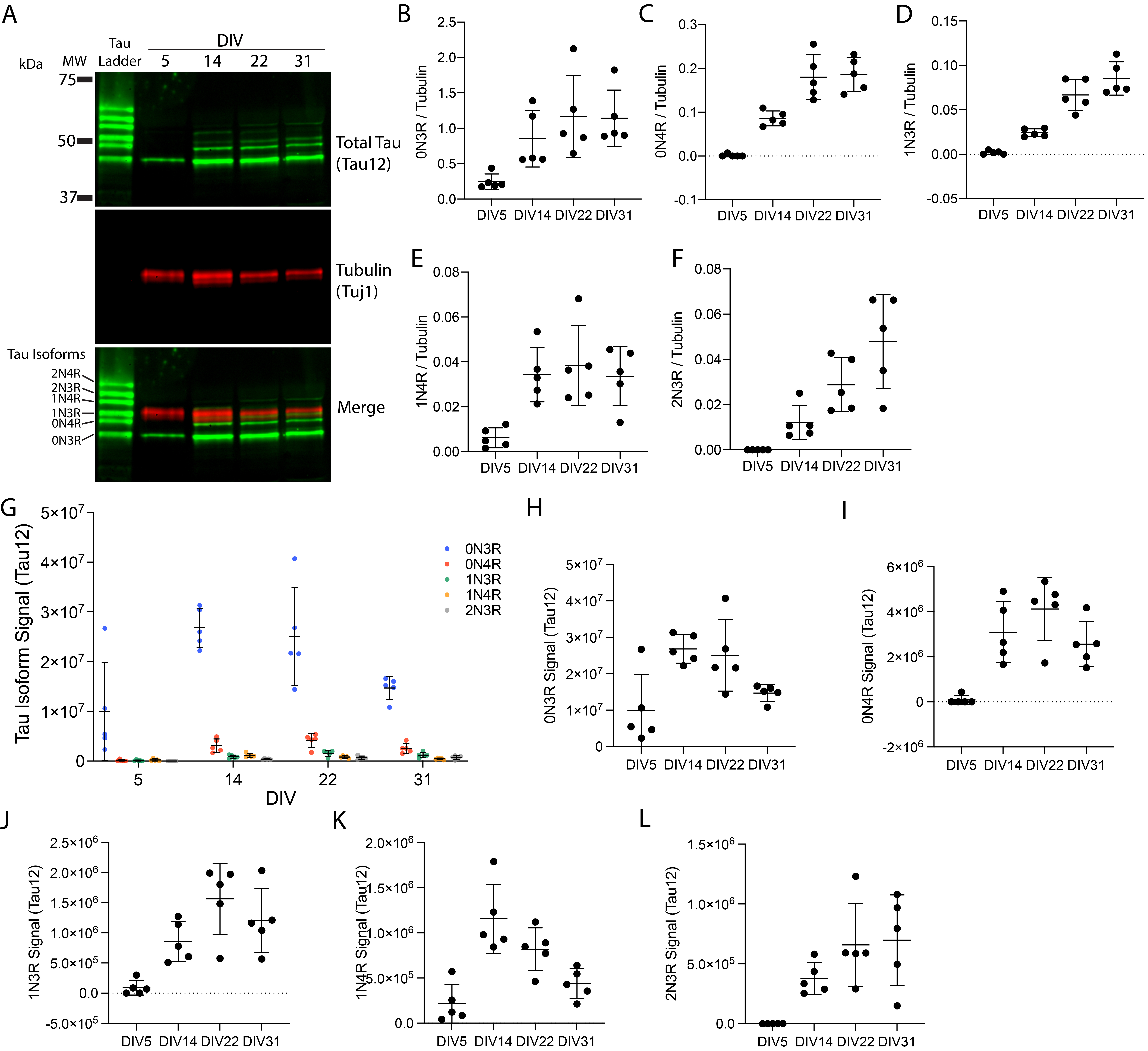


Supplementary Fig. 1. Expression of 4R and 3R human tau isoforms increases over time in MAPT-KI primary hippocampal cultures. A) Representative western blot of culture lysates collected on DIV5, 14, 22, and 31, probed for total tau (Tau12 antibody; green) and β-III tubulin (Tuj1 antibody; red). A recombinant human tau ladder was included for reference. Uncropped versions of the representative blot are shown in Supplementary Fig. 14A. B-F). The intensity of each tau isoform band was quantified and normalized to β-III tubulin bands (Tuj1 antibody). G-L) Signal for each tau isoform (Tau12 antibody) is displayed without β-III tubulin normalization. The data are shown on one graph to visualize relative differences across isoform at each time point (G), as well as graphs of individual isoforms to better highlight time-dependent changes for each isoform (H-L). Data are mean ±SD. N = 5.


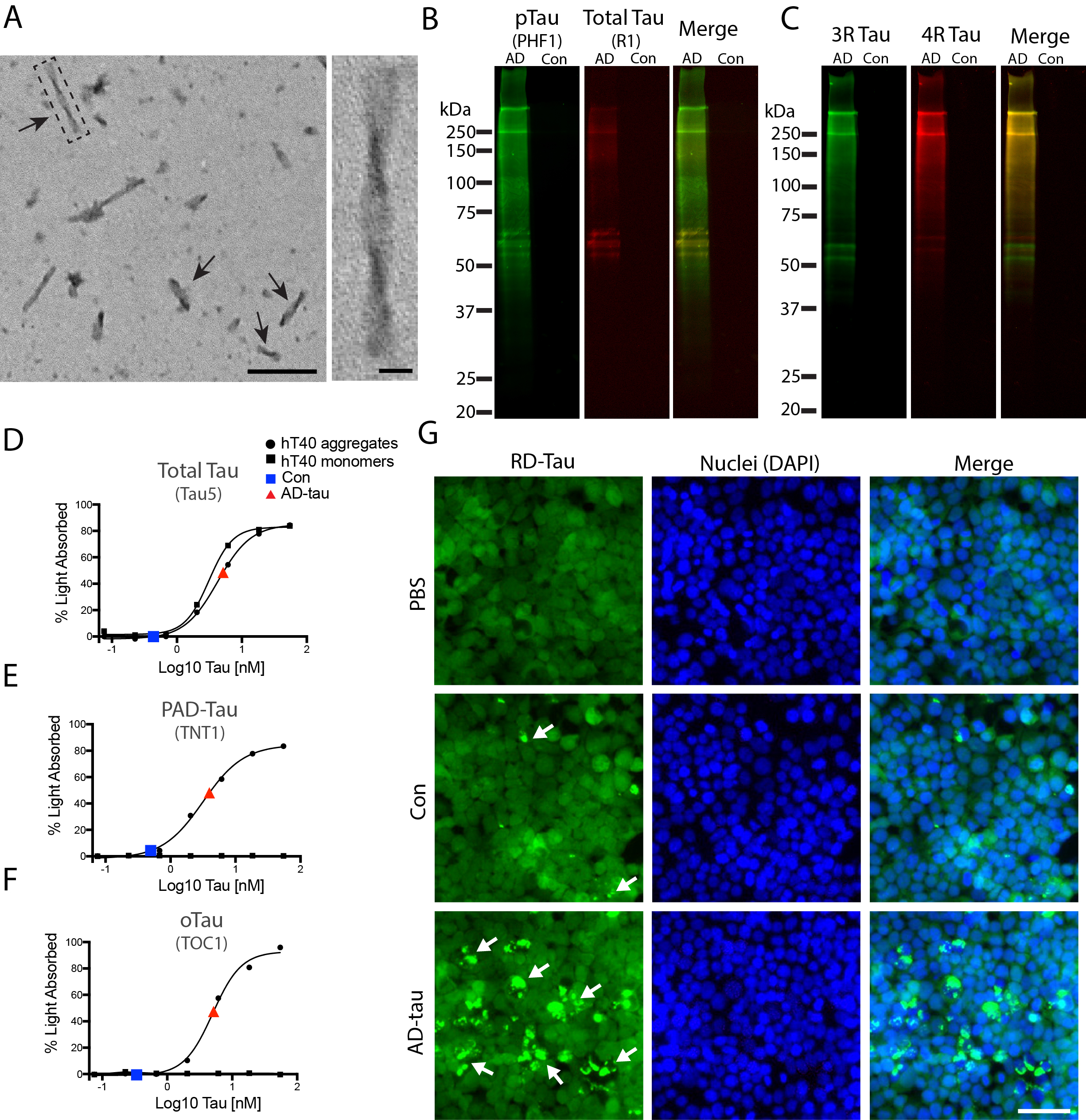


Supplementary Fig. 2. AD brain-derived tau samples contain known disease-associated pathological tau species and are seed-competent in Tau RD P301S Biosensor cells. A) Representative TEM photomicrograph of AD-tau before sonication confirms the presence of paired helical filaments (arrows). Scale bar = 200 nm; inset 25 nm. B) Western blot of AD-tau or Con samples probed for total tau (R1 antibody; red) and phospho-tau (pTau; PHF1 antibody; green). C) Western blot of AD-tau or Con samples probed with 3R tau isoform (green) and 4R tau isoform (red) antibodies. Uncropped versions of the blots in B and C are shown in Supplementary Fig. 14B. D-F) Sandwich ELISAs of AD-tau or Con samples to measure total tau (Tau5 antibody; D), PAD-exposed tau (PAD-Tau; TNT1 antibody; E), or oligomeric tau (oTau; TOC1 antibody; F). G) Tau RD P301S biosensor cells that express fluorescently tagged RD P301S tau (RD-Tau; green) show low or high levels of aggregated tau puncta (arrows) when treated with Con or AD-tau (2.8 nM tau) seeding material, respectively. PBS treated cells do not form tau aggregates. Cells were counterstained with a nuclear counterstain (DAPI, blue). Scale bar = 50 µm.


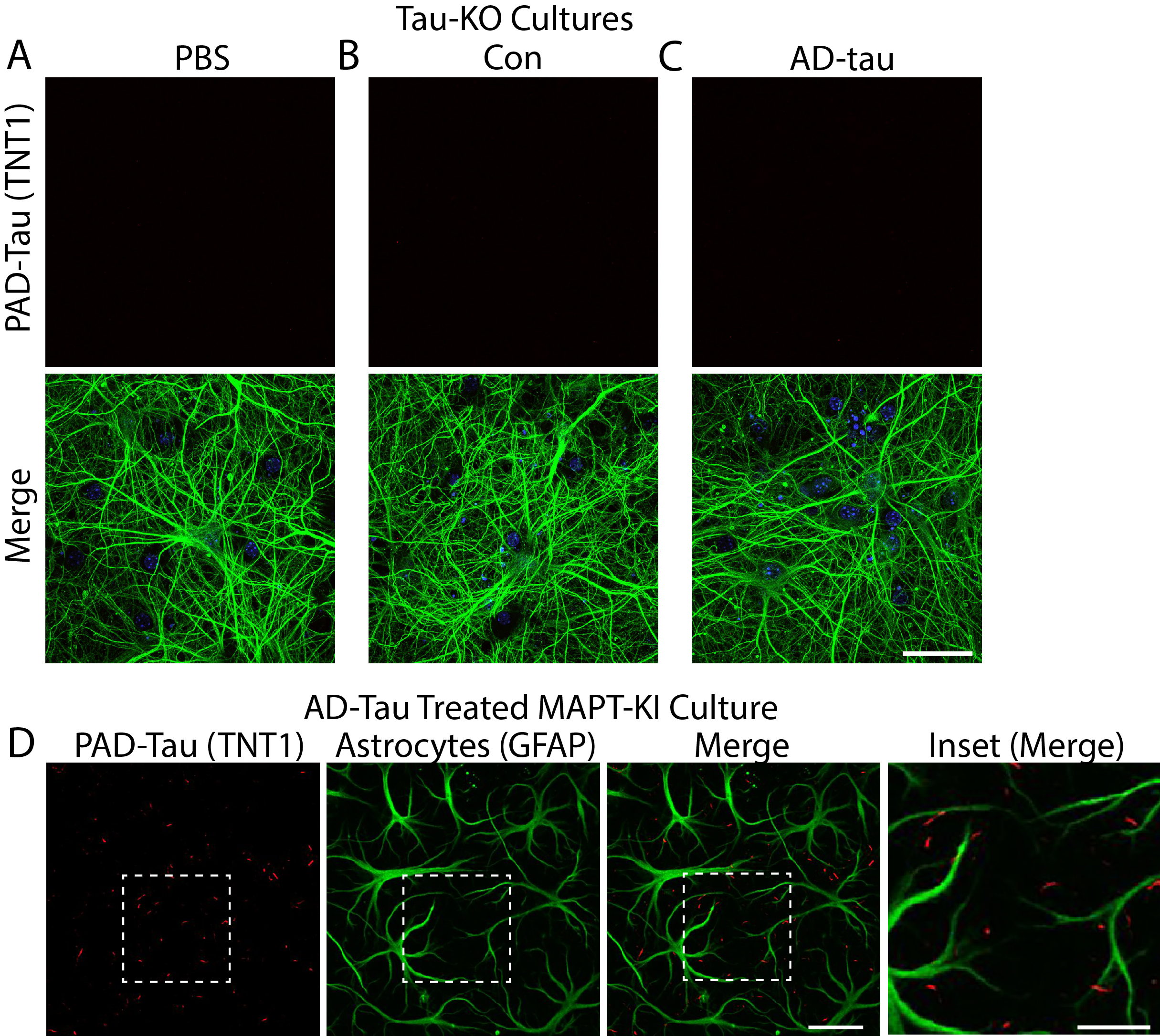


Supplementary Fig. 3. PAD-exposed tau inclusions are absent in AD-tau treated Tau-KO primary neurons and in MAPT-KI astrocytes at 28d post-treatment. A-C) PAD-exposed tau inclusions (PAD-Tau; TNT1 antibody; top panels), are not present in Tau-KO primary cultures treated with PBS (A), Con (B) or AD-tau (C). The merged images include immunolabeling with PAD-Tau (red), β-III tubulin (green) and a nuclear counterstain (DAPI, blue). Scale bar = 50 µm. D) PAD-exposed tau (PAD-Tau; red) inclusions are absent in astrocytes, as indicated with GFAP (green), from MAPT-KI primary hippocampal cultures treated with AD-tau. Scale bar = 50 µm; inset scale bar = 25 µm. N = 3.


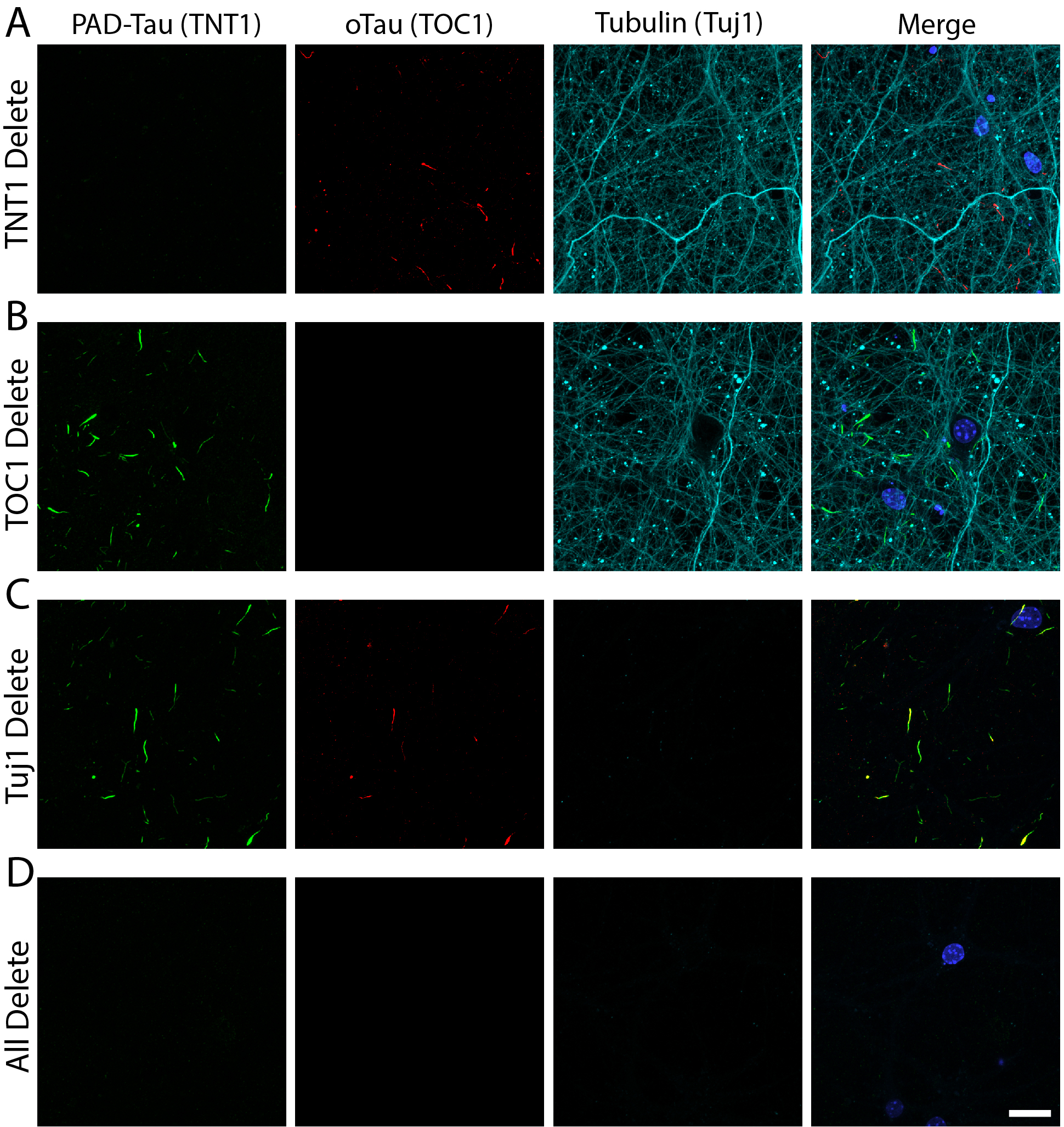


Supplementary Fig. 4. Primary delete control staining for PAD-exposed tau, oligomeric tau and β-III tubulin. A-D) Primary delete control labeling in Con or AD-tau treated neurons for PAD-exposed tau (PAD-Tau; TNT1 antibody; green), oligomeric tau (oTau; TOC1 antibody; red) and β-III tubulin (Tuj1 antibody; cyan). The lack of signal in the primary antibody deletes confirms that these stains did not cross react with each other in all cases. Scale bars = 20 µm. N = 3.


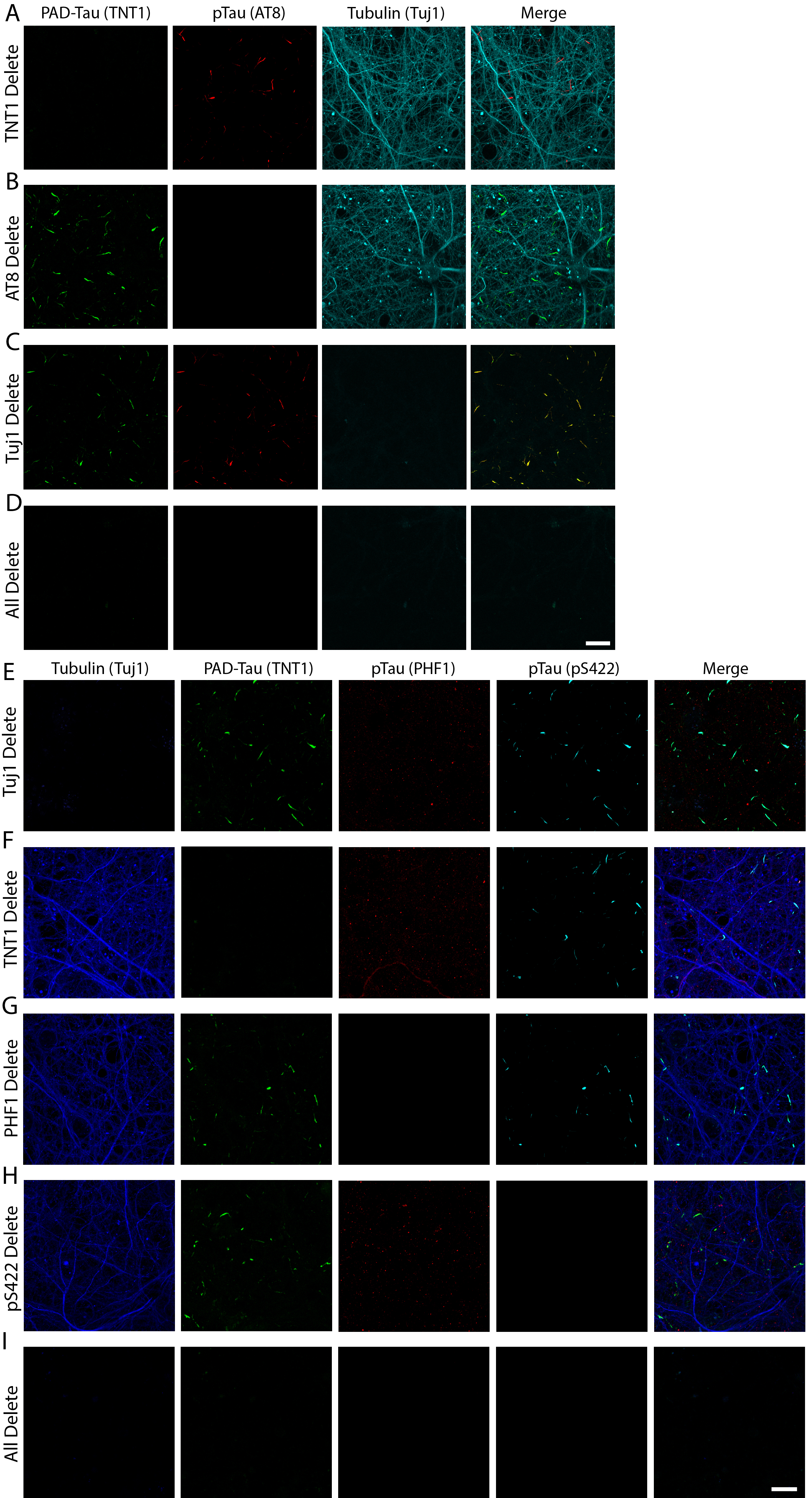


Supplementary Fig. 5. Primary delete control staining for PAD-exposed tau, phospho-Tau and β-III tubulin. A-D) Primary delete control labeling in Con or AD-tau treated neurons for PAD-exposed tau (PAD-Tau; TNT1 antibody; green), phospho-AT8 tau (pTau; AT8 antibody; red) and β-III tubulin (Tuj1 antibody; cyan). E-I) Primary delete control labeling in Con or AD-tau treated neurons for β-III tubulin (blue), PAD-exposed tau (PAD-Tau; green), phospho-PHF1 tau (pTau; PHF1 antibody; red), and phospho-S422 tau (pTau; pS422 antibody; cyan). The lack of signal in the primary antibody deletes confirms that these stains did not cross react with each other in all cases. Scale bars = 20 µm. N = 3.


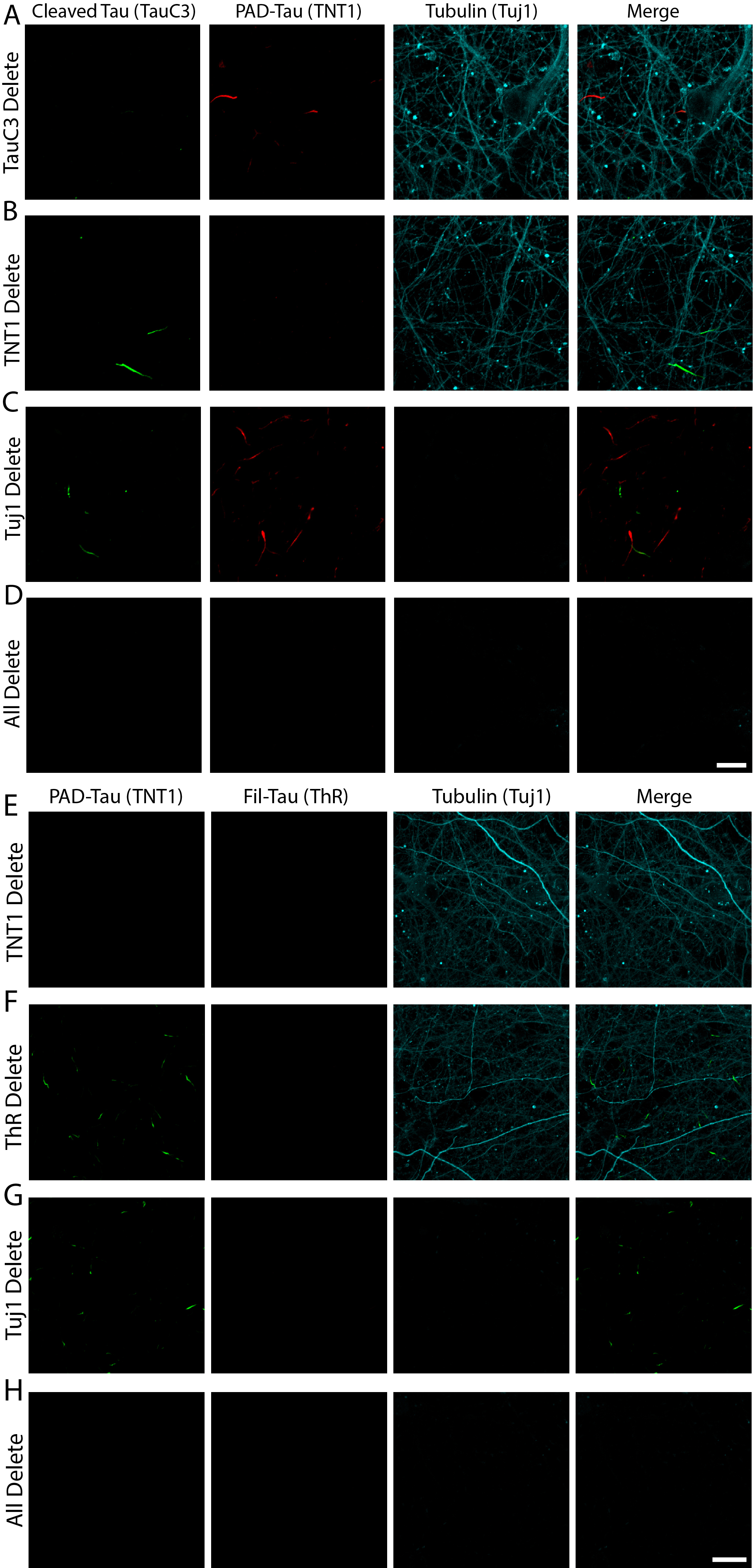


Supplementary Fig. 6. Primary delete control staining for PAD-exposed tau, cleaved tau and filamentous tau. A-D) Primary delete control labeling in Con or AD-tau treated neurons for caspase cleaved tau (TauC3 antibody; green), PAD-exposed tau (PAD-Tau; TNT1 antibody; red), and β-III tubulin (Tuj1 antibody; cyan). E-H) Primary delete control labeling in Con or AD-tau treated neurons for PAD-exposed tau (PAD-Tau; green), filamentous tau (Fil-Tau; ThR dye; red), and β-III tubulin (cyan). The lack of signal in the primary antibody deletes and with ThR omission confirms that these stains did not cross react with each other in all cases. Scale bars = 20 µm. N = 3.


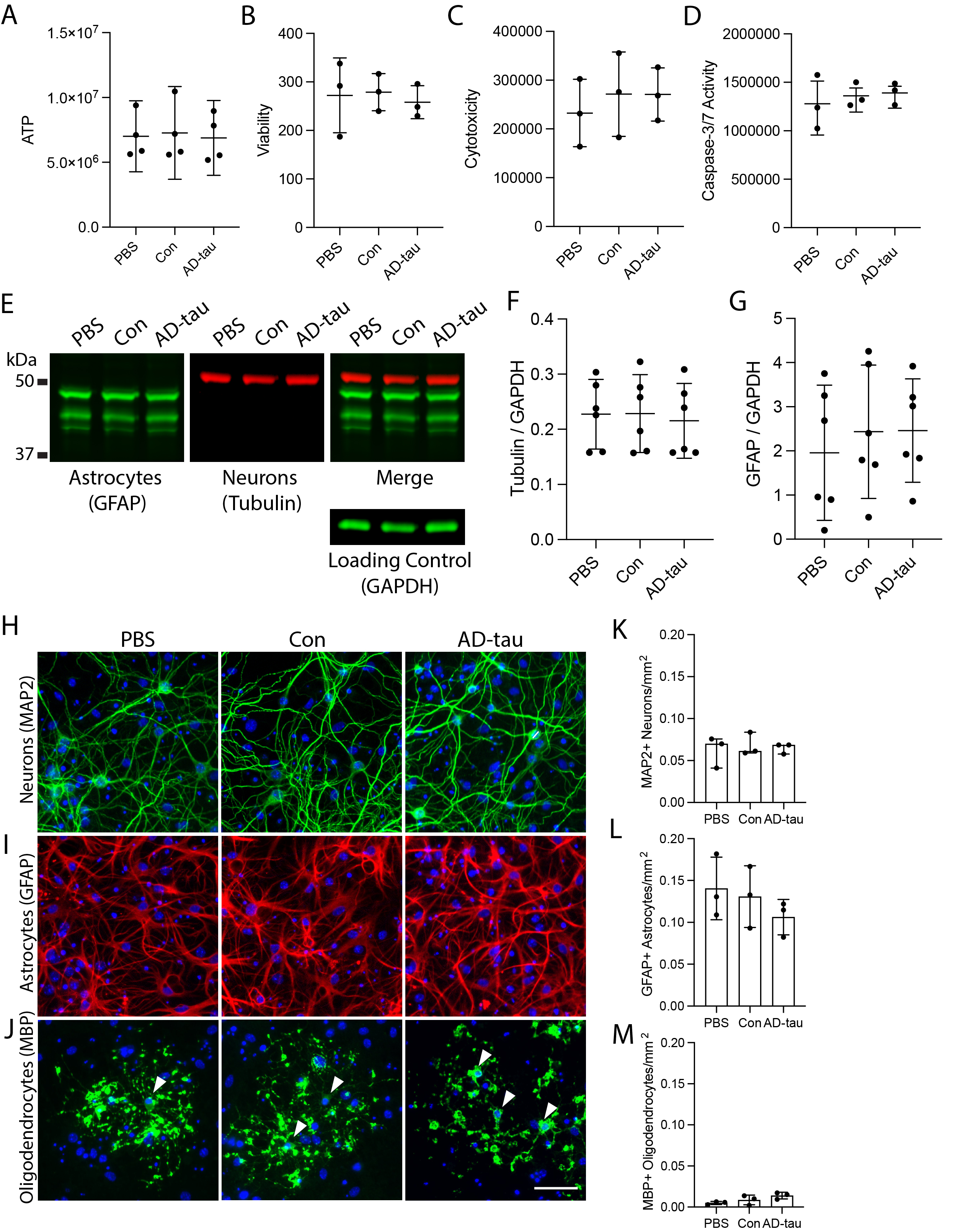


Supplementary Fig. 7. Tau seeding did not affect cell culture viability out to 28d post-treatment. A-D) Primary MAPT-KI hippocampal cultures were treated with PBS, Con, or AD-tau (28 nM) on DIV5 and showed no differences in cell viability at DIV33 with the CellTiter-Glo assay (A; ATP levels; *F*_(2,9)_ = 0.04135, p = 0.9597), or at DIV31 with the ApoTox-Glo assay that measures viability (B; *F*_(2,6)_ = 0.1169, p = 0.8917), cytotoxicity (C; *F*_(2,6)_ = 0.2899, p = 0.7583), and caspase-3/7 activity (D; *F*_(2,6)_ = 0.2853, p = 0.7614). E-G) Western blots (E) of culture lysates collected at DIV31 probed for β-III tubulin (Tuj1 antibody; red) as a neuron marker (F; *F*_(2,15)_ = 0.0771, p = 0.9262), glial fibrillary acidic protein (GFAP; green) as an astrocyte marker (G; *F*_(2,15)_ = 0.2658, p = 0.7701), and GAPDH (green) as a loading control. Uncropped versions of the representative blot are shown in Supplementary Fig. 14C. The membranes were cut just below 37 kDa and the top probed with GFAP and Tuj1 antibodies, while the bottom was probed with GAPDH antibody. H-J) Representative images of neurons (H; MAP2 antibody, green), astrocytes (I; GFAP antibody, red) and oligodendrocytes (J; MBP antibody, green) in primary MAPT-KI hippocampal cultures treated with PBS, Con, or AD-tau on DIV5 and fixed at DIV33. Nuclei were stained with DAPI (blue). Scale bar = 50 µm. K-M) Neurons (K; H = 0.2689, p = 0.9071), astrocytes (L; *F*_(2,3)_ = 0.0711, p = 0.4655l) and oligodendrocytes (M; *F*_(2,3)_ = 3.257, p = 0.1102l) were manually counted and were not statistically different among groups. Notably, oligodendrocyte numbers increased by 71% in Con and 176% in AD-tau cultures when compared to PBS cultures, but these differences were not statistically significant due to high variability. Three images were used for each independent experimental replicate. The data in A-D, F-G, and L-M are mean ±SD and were compared using one-way ANOVA. The data in K are median ±interquartile range and were compared using the nonparametric Kruskal-Wallis ANOVA. Significance in all cases was defined as p ≤ 0.05. N = 4 (A); N = 3 (B-D and K-M); N = 6 (F-G).


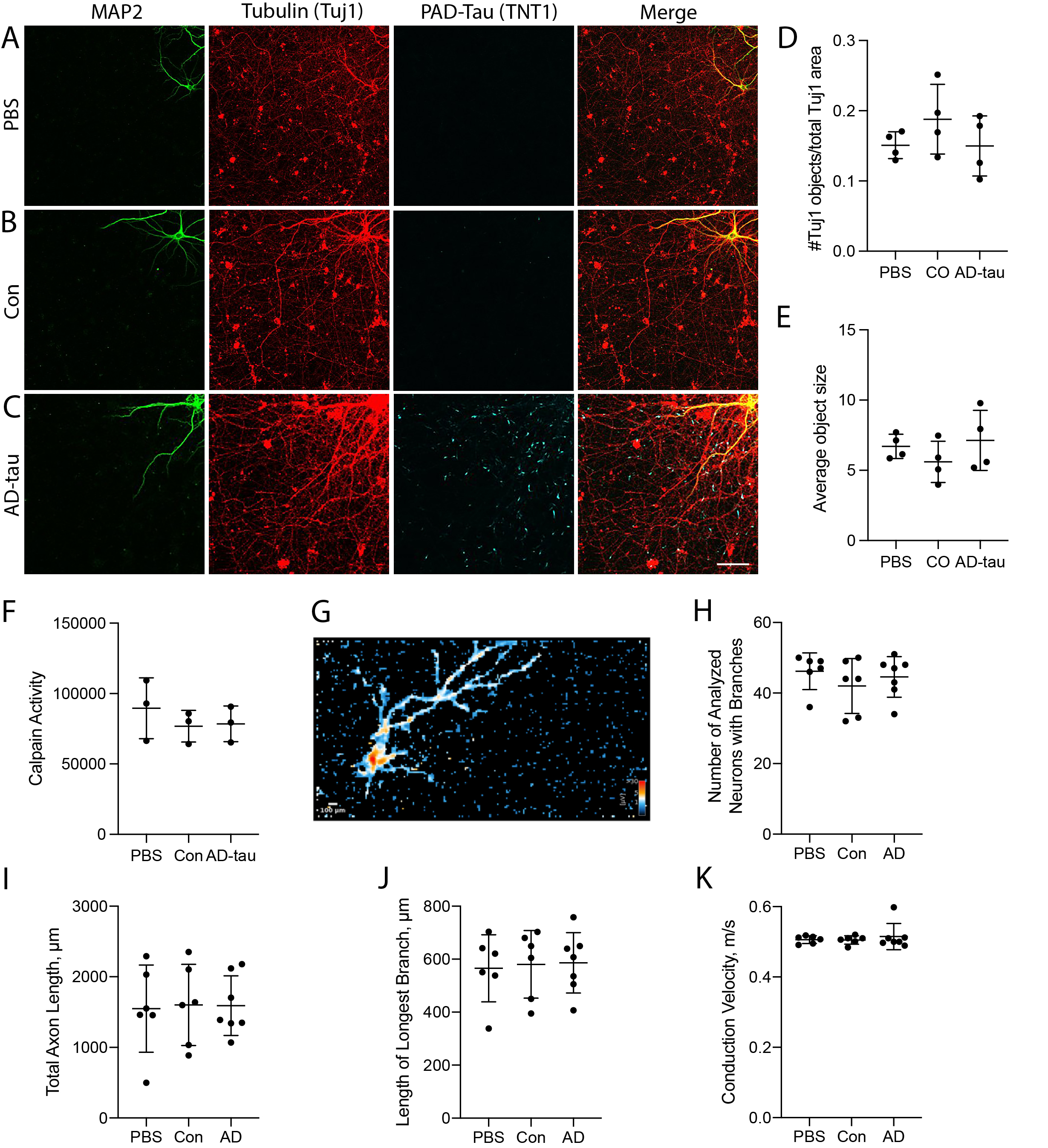


Supplementary Fig. 8. Overt axonal degeneration was not observed in AD-tau treated MAPT-KI primary neurons. A-C) Representative images of the axon outgrowth in PBS, Con, and AD-tau treated cultures stained for a neuronal somatodendritic marker (MAP2 antibody; green), a neuronal soma, dendrite and axon marker (β-III tubulin; Tuj1 antibody; red), and PAD-exposed tau (PAD-Tau; TNT1 antibody; cyan). Scale bar = 50 µm. D-E) There were no differences in the number of β-III tubulin objects normalized to total β-III tubulin area (D; *F*_(2,9)_ = 1.219., *p* = 0.3400) or mean β-III tubulin object size (E; *F*_(2,9)_ = 0.9936, *p* = 0.4075; N = 4). F) Calpain activity was not different across groups (*F*_(2,6)_ = 0.5674, *p* = 0.5947; N = 3). G-K) Microelectrode array (MEA) analyses indicated no axonal degeneration among the treatment groups. Axonal arborizations were tracked using the Axonal Tracking Assay and a representative arborization for a single neuron is shown (G). H) The number of analyzed cells with branches was similar across groups (H = 1.099, p = 0.5964; N = 6). I) There were no differences in the mean total axonal length (H = 0.1520, p = 0.9379; N = 6) or the length of the longest axonal branch (J; *F*_(2,15)_ = 0.03201, *p* = 0.9686; N = 6). K) Treatment conditions did not affect the mean action potential conduction velocity (H = 0.1089, p = 0.9453; N = 6). The data in D-F and J are mean ±SD and were compared using one-way ANOVA. The data in H, I, and K are median ±interquartile range and were compared using the nonparametric Kruskal-Wallis ANOVA. Significance in all cases was defined as p ≤ 0.05. N = 4 (D,E); N = 3 (F); N = 6 (H-K); each N represents a separate primary neuron harvest.


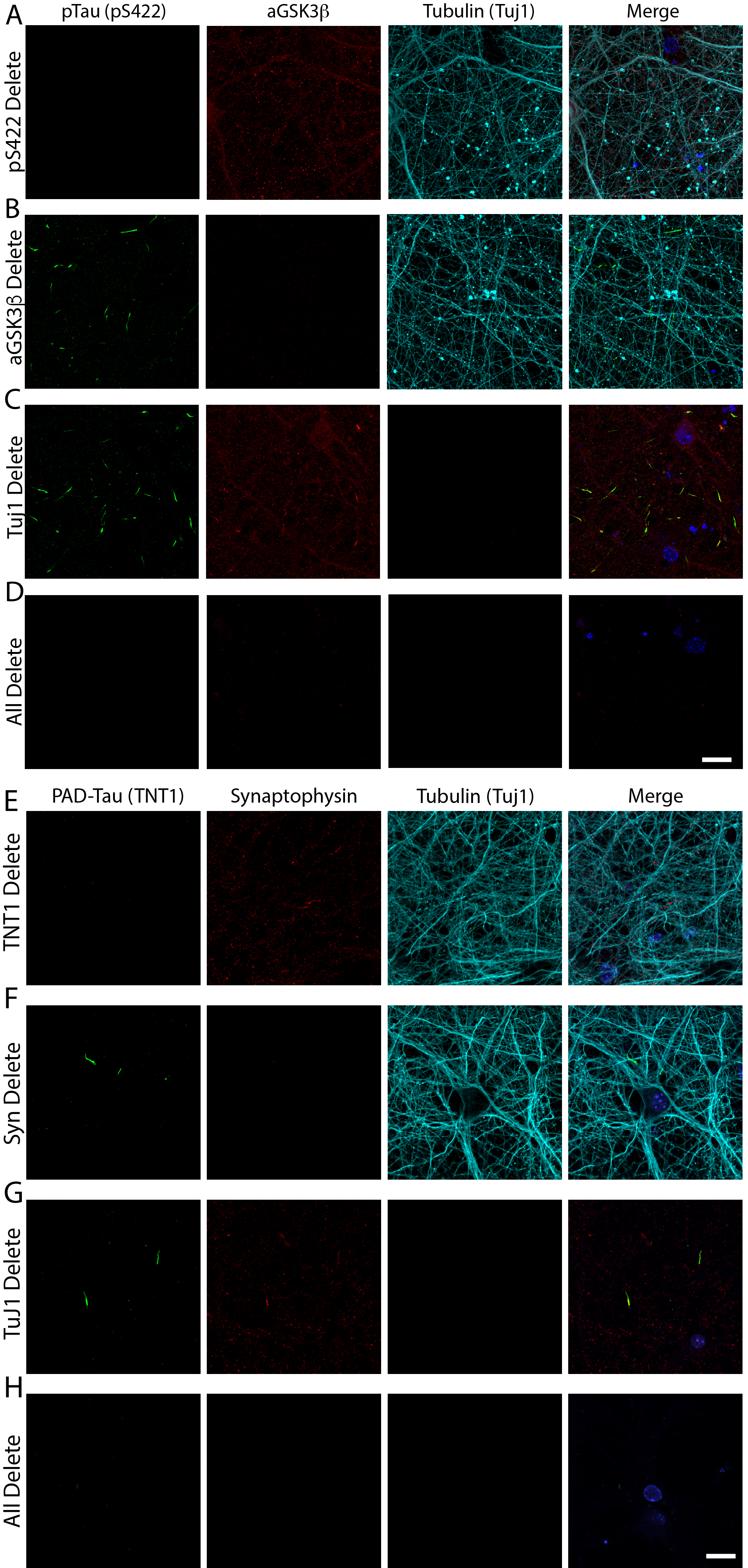


Supplementary Fig. 9. Primary delete control staining for PAD-exposed tau, active GSK3β, synaptophysin and β-III tubulin. A-D) Primary delete control labeling in Con or AD-tau treated neurons for phospho-S422 tau (pS422 antibody; green), active GSK3β (aGSK3β; non-phospho-Ser9 GSK3β; red) and β-III tubulin (Tuj1 antibody; cyan). The merge also includes a nuclear counterstain (DAPI, blue). E-H) Primary delete control labeling in Con or AD-tau treated neurons for PAD-exposed tau (PAD-Tau; TNT1 antibody; green), synaptophysin (synaptophysin antibody; red) and β-III tubulin (cyan). The merge also includes a nuclear counterstain (DAPI, blue). The lack of signal in the primary antibody deletes confirms that these stains did not cross react with each other. Scale bar = 20 µm. N = 3.


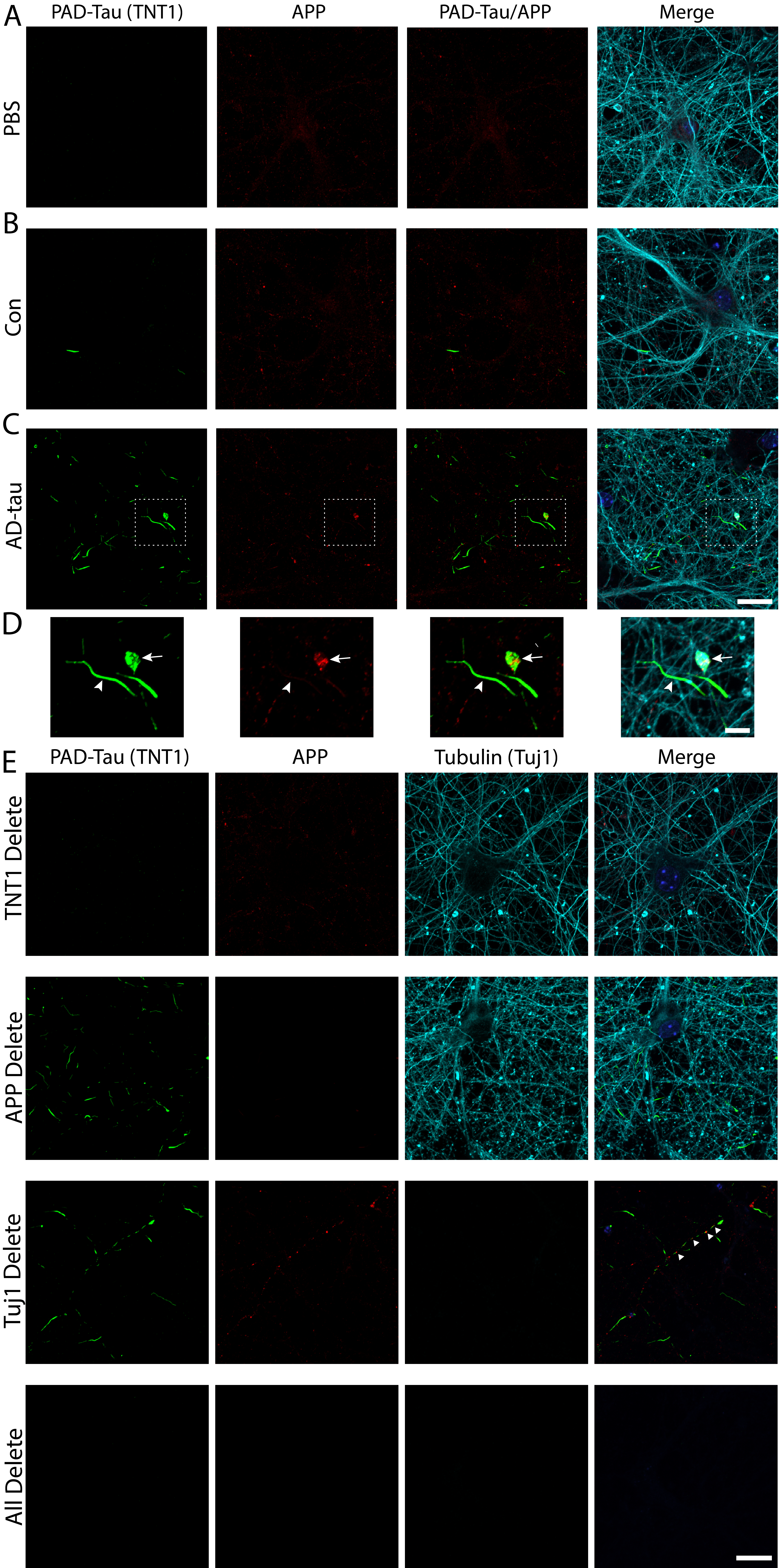


Supplementary Fig. 10. Amyloid precursor protein (APP) cargoes co-accumulate with few pathological tau inclusions. Cultures were treated on DIV5 with PBS, Con, or AD-tau and fixed at DIV21 (16d post-treatment) for immunostaining. A-C) APP (APP antibody; red) showed relatively low levels of colocalization with PAD-exposed tau inclusions (PAD-Tau; TNT1 antibody; green) but was present in axonal swellings in AD-tau neurons (C; arrow). Scale bar = 20 µm. D) Insets show colocalization between PAD-tau and APP (arrow) in a spheroid and inclusions without colocalization between PAD-tau and APP (arrowhead). Scale bar = 5 μm. E) Primary delete control labeling in AD-tau cultures confirms that the TNT1 (green), APP (red), and Tuj1 (cyan) antibody stains did not cross react with each other. Note, an example of APP appearing to associate with the ends of some PAD-exposed tau positive inclusions is shown in Tuj1 Delete (arrowheads). Scale bar = 20 µm. N = 3.


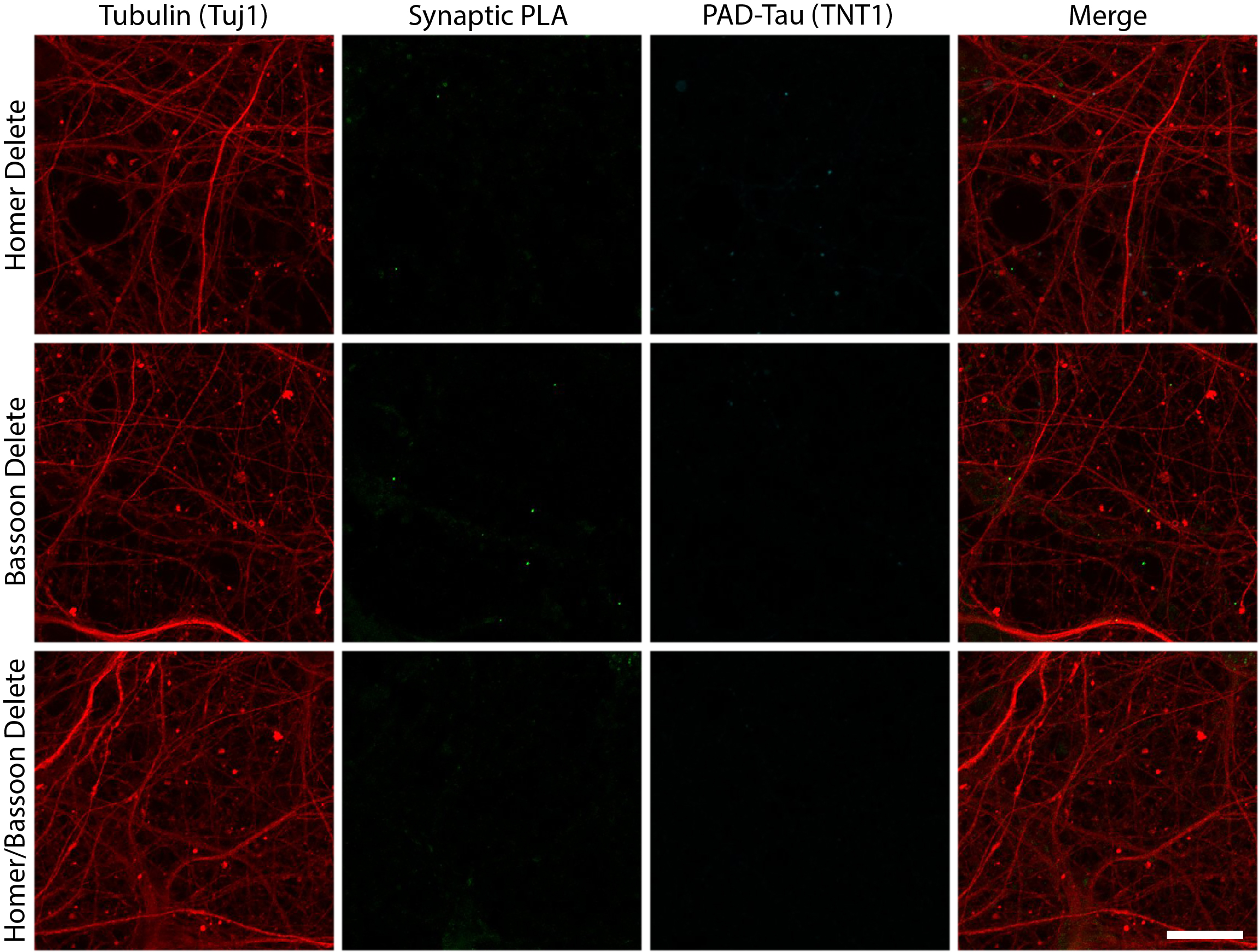


Supplementary Fig. 11. Proximity ligation assay (PLA) primary delete control staining. Untreated cultures were used for the primary delete control labeling. PLA was followed by immunostaining for β-III tubulin (Tuj1 antibody; red) and PAD-exposed tau (TNT1 antibody; cyan). Omission of the homer primary antibody, the bassoon primary antibody, or both showed very low PLA signal (green). TNT1 signal was not observed in the untreated cells. Scale bar = 20 µm.


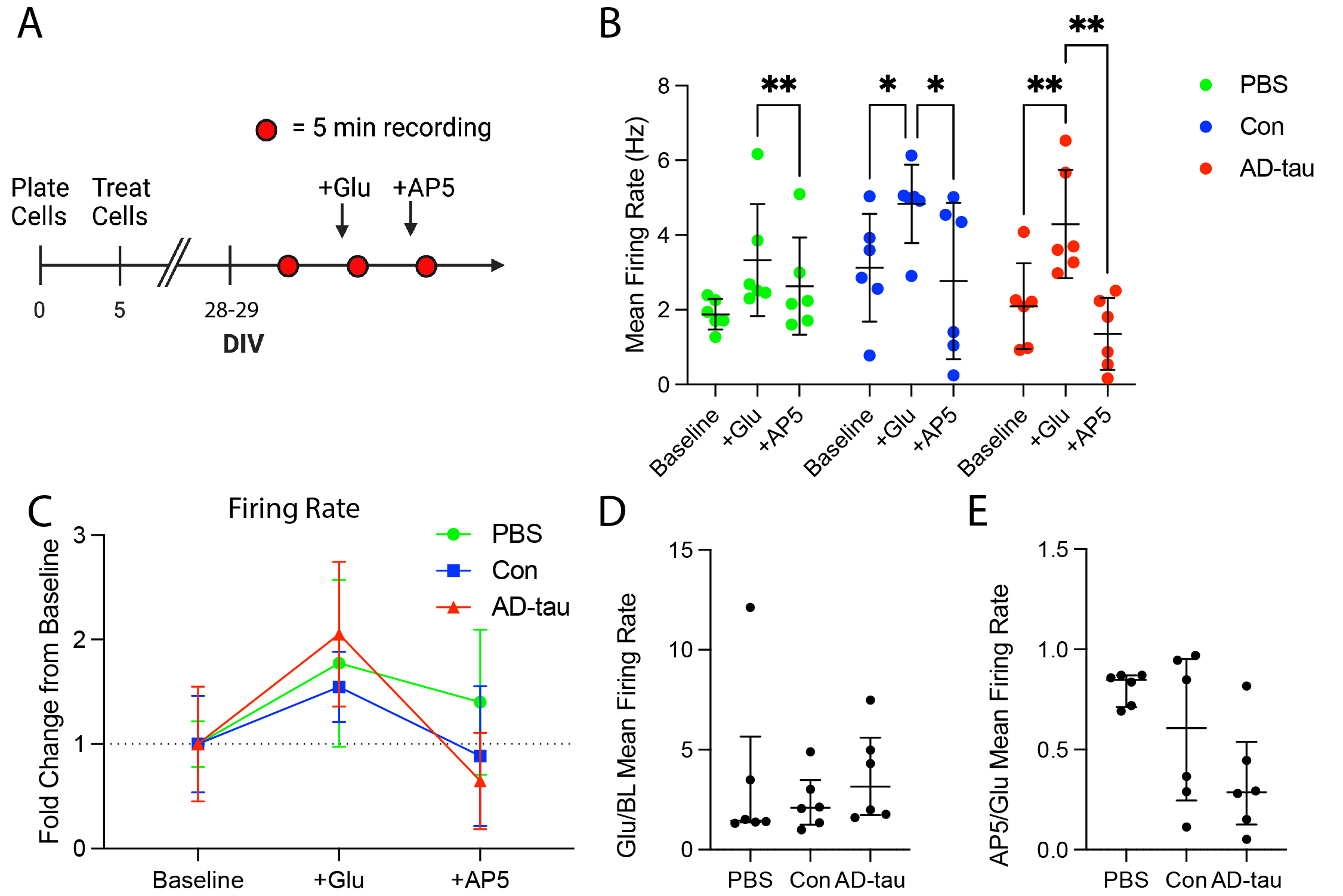


Supplementary Fig. 12. AD-tau treated cultures do not exhibit significant changes in mean firing rate when compared to PBS or Con cultures. A) MAPT-KI cultures were plated on high-density microelectrode array chips and treated with PBS, Con, or AD-tau on DIV5. On DIV28-29, three consecutive 5-minute neuronal activity recordings (red circles) were acquired: basal activity, after addition of glutamate (+Glu; 20 µM final concentration) and after addition of NMDAR antagonist (+AP5; 5 µM final concentration). B) Within groups, +Glu increased firing rate in PBS cultures (77% increase) compared to baseline, but this did not reach statistical significance (p = 0.13). On the other hand, +Glu significantly increased firing rate in both Con (54% increase) and AD-tau (105% increase) cultures from baseline. Within groups, +AP5 significantly reduced firing rate compared to +Glu in PBS (21% decrease), Con (43% decrease) and AD-tau (68% decrease) cultures. Comparisons across PBS, Con and AD-tau cultures showed there were no differences in the mean firing rate at baseline, after the addition of Glu, or after the addition of AP5 (Recording phase (baseline, +Glu, or +AP5): *F*_(2, 45)_ = 2.104 p < 0.0001; Treatment: (PBS, Con, or AD-tau) *F*_(2, 45)_ = 1.677 p = 0.2201; Interaction: *F*_(4, 45)_ = 2.104 p = 0.1050; PBS +Glu vs. +AP5 p = 0.0031; Con BL vs. +Glu: p = 0.0426, +Glu vs. +AP5: p = 0.0485; AD-tau BL vs. +Glu: p = 0.0016, +Glu vs. +AP5: p = 0.0059). The data in B are mean ±SD and were compared using two-way repeated measures ANOVA with Tukey’s multiple comparisons test. *p ≤ 0.05, **p ≤ 0.01. C) The fold-change from baseline to +Glu and from baseline to +AP5 were calculated (data are mean ±SD). All three groups showed increased firing rates in response to Glu when compared to baseline (PBS = 1.77-fold; Con = 1.55-fold; AD-tau = 2.05-fold). All three groups showed reduced firing rates from Glu after AP5 treatment. When compared to baseline, PBS cultures showed a 1.40-fold change, while Con cultures were 0.88-fold and AD-tau cultures were 0.65-fold reduced from baseline. After both treatments, the changes in firing rate were greatest in AD-tau cultures. D-E) The magnitude of the response to Glu (+Glu/Baseline; D) and then to AP5 (+AP5/+Glu; E) was quantified. There was no significant treatment effect on the response to +Glu (D; *H* = 1.766, p = 0.4317) or the response to +AP5 (E; *H* = 5.632, p = 0.0537). However, there was a trend showing a greater change from +Glu to +AP5 in the AD-tau cultures compared to the PBS controls (E; p = 0.06). The data in D and E are median ±interquartile range and were compared using the Kruskal-Wallis ANOVA test, significance was defined as p ≤ 0.05; N = 6 for all experiments.


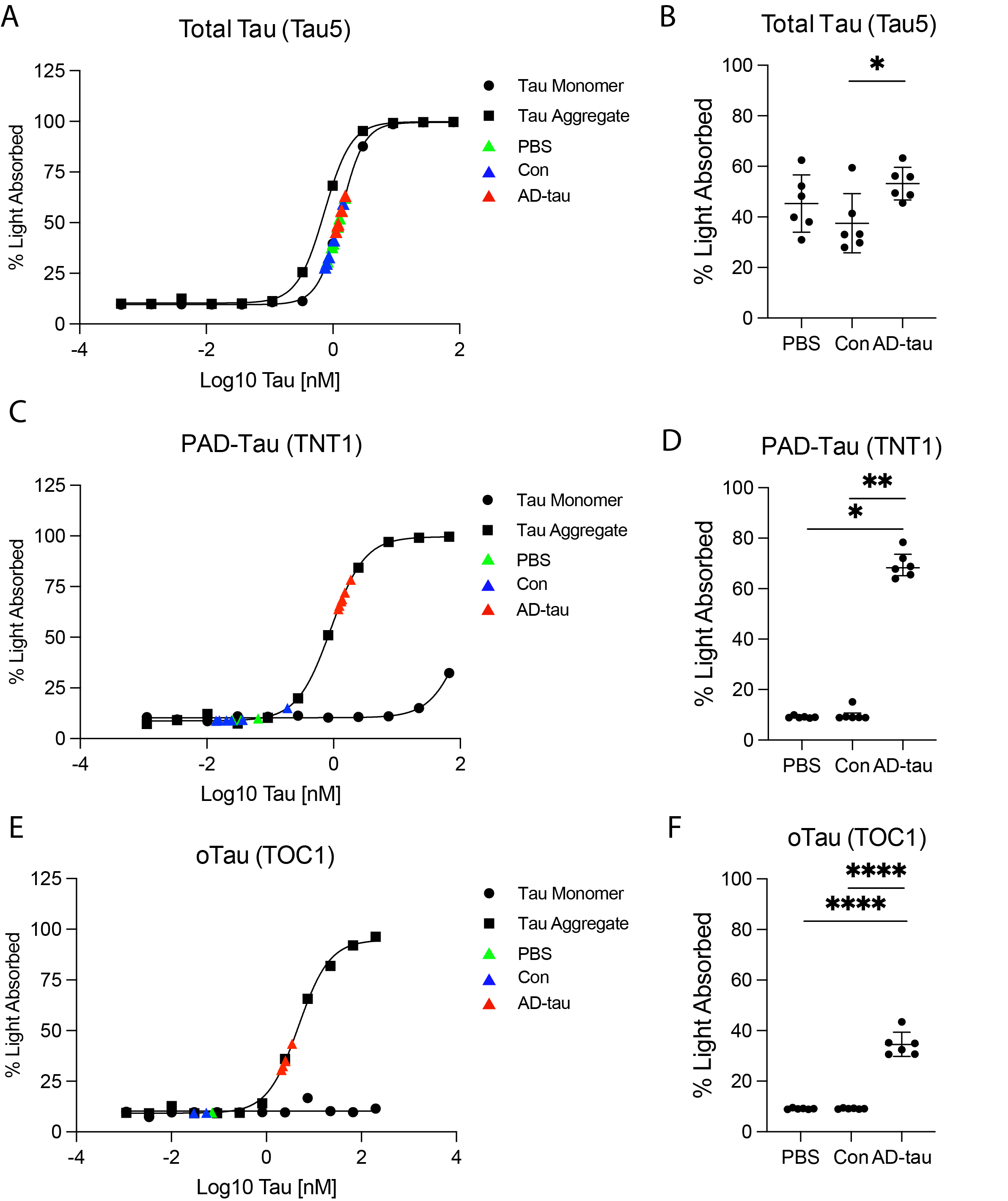


Supplementary Fig. 13. AD-tau treated cultures used in microelectrode array (MEA) experiments contain pathological tau species. After collecting MEA recordings, lysates were collected, and the presence of pathological tau species was quantified using sELISA. Samples were analyzed for total tau (Tau5 antibody; A-B), PAD-exposed tau (PAD-Tau; TNT1 antibody; C-D), or oligomeric tau (oTau; TOC1 antibody; E-F) and detected with a rabbit polyclonal pan tau antibody (R1 antibody). Recombinant tau monomer and aggregate (2N4R isoform) standard curves were included for each assay (A, C, E). Similar levels of total tau (Tau5 assay) were detected in all cultures but there was a significant increase in signal in the AD-tau cultures compared to Con-treated cultures (A-B; *F*_(2,15)_ = 3.616, p = 0.0523; *p = 0.0145). C-D) Levels of pathological PAD-exposed tau (TNT1 assays) were significantly increased in AD-tau treated cultures (H = 11.46, p = 0.0005; *p = 0.0072; **p = 0.0145). E-F) Oligomeric tau (TOC1 assays) was also detected in AD-tau treated cultures but not PBS or Con treated cultures (*F*_(2,15)_ = 168.4, p < 0.0001; ****p < 0.0001). In B and F, the data are mean ±SD and were compared using one-way ANOVA with Tukey’s multiple comparisons test. In D, the data are median ±interquartile range and were compared using the nonparametric Kruskal-Wallis ANOVA with Dunn’s multiple comparisons test. N = 6.


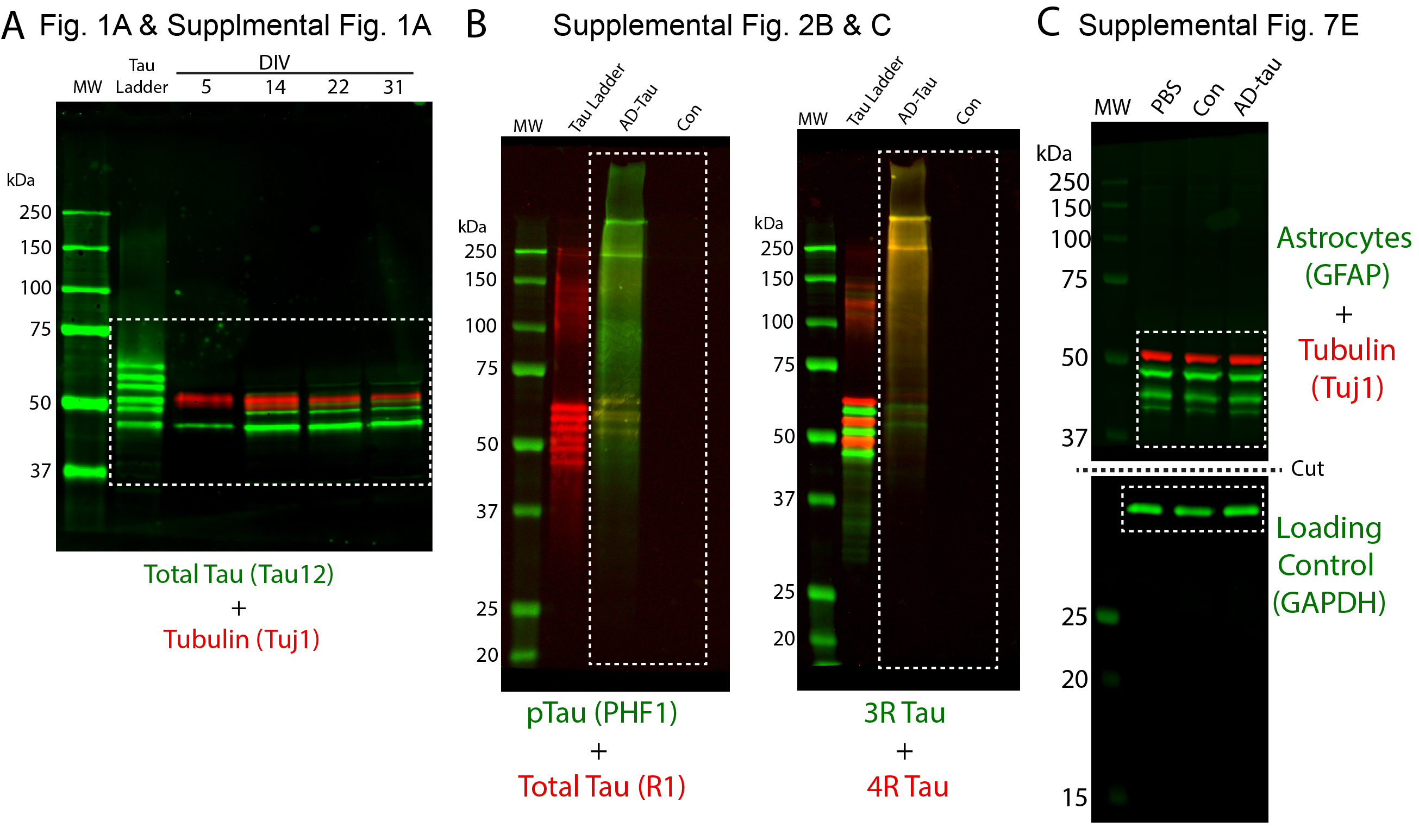


Supplementary Fig. 14. Full versions of representative immunoblots. A-C) Immunoblots from Fig. 1A and Supplementary Fig. 1A (A), Supplementary Fig. 2B and C (B), and Supplementary Fig. 7E (C) are shown with the dashed white box indicating the cropped portion used in the respective figures. In C, membranes that were cut to allow probing with multiple antibodies are indicated in the Methods section. See main and supplementary methods for immunoblotting details.

**Supplementary References**

1. Combs B, Tiernan CT, Hamel C, Kanaan NM: **Production of recombinant tau oligomers in vitro.** *Methods Cell Biol* 2017, **141:**45-64.
